# Near‐Unity Photothermal Conversion in Bimetallic M_44_ and M_81_ Nanoclusters Enables NIR‐Driven Photo‐Thermo‐Electricity Generation

**DOI:** 10.1002/advs.78061

**Published:** 2026-09-30

**Authors:** Avirup Sardar, Abhrojyoti Mazumder, Weijie Ji, Guiying He, Yitong Wang, Lianshun Luo, Sihan Chen, Rongchao Jin

**Affiliations:** ^1^ Department of Chemistry Carnegie Mellon University Pittsburgh USA

**Keywords:** alloy nanoclusters, atomically precise nanoclusters, energy conversion, near‐infrared, photothermal

## Abstract

More than half of the solar energy lies in the near‐infrared (NIR) range; however, utilizing a significant amount of energy in this region remains largely inaccessible in conventional photovoltaic technologies. Here, we establish quantum‐sized nanoclusters (NCs) as a new class of NIR‐driven photo‐thermo‐electric transducers via light‐to‐heat‐to‐electricity cascade conversion. Two alkynyl‐protected alloy NCs, namely, Au_24_Ag_20_ (M_44_) and Au_43_Ag_38_ (M_81_), exhibit NIR‐II photoluminescence, being rare among the NCs. More strikingly, the NC solutions deliver record photothermal conversion efficiencies of 69% and 91% with M_44_ and M_81_, respectively, which are the highest reported for atomically precise nanomaterials to date, together with their exceptional solid‐state temperature rise of more than 200°C under 808 nm irradiation for thermoelectric generation (TEG) while maintaining photostability. The nanocluster absorption profiles overlap well with the solar spectrum, suggesting the potential of such NCs in converting sunlight to thermoelectricity. The current work will drive the future design of next‐generation NC‐based devices that are capable of harvesting the NIR solar spectrum for light‐to‐heat‐to‐electricity conversion and other photonic energy harvesting technologies.

## Graphical abstract

Atomically precise nanoclusters of Au/Ag alloys are explored as high‐performance photothermal materials, delivering record‐high photothermal efficiencies (up to 91%). Photo‐thermo‐electricity is further demonstrated by integrating nanocluster films with thermoelectric devices.

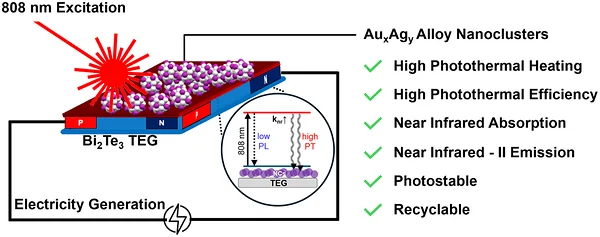

## 1 Introduction

Materials exhibiting strong near‐infrared (NIR) activity have attracted tremendous research interest due to their potential applications spanning across various fields [1, 2, 3, 4, 5]. While NIR‐emitting materials are highly attractive in the fields of deep‐tissue bioimaging, optical security, and secure telecom technologies, NIR‐absorbing materials have become a cornerstone for advanced photothermal therapy, optical filtering, and next‐generation solar energy harvesting [6, 7].

Recently, a new class of nanomaterials—atomically precise nanoclusters (APNCs) of metals—has emerged as an ideal candidate for these applications due to their controllable absorption and emission spanning the NIR to the short‐wave infrared (SWIR) [8, 9, 10, 11]. Their monodispersity, <2 nm dimensions, quantum confinement effect, and well‐defined atomic structures allow for the exploration of optoelectronic properties. Such functionality can be controlled at the atomic level by varying the size (i.e., the precise number of atoms), composition (e.g., doping or alloying), or the organic ligand shell, which are hard to attain in conventional nanoparticles [12, 13, 14, 15, 16, 17]. Considerable research has been done by various groups to expand the library of metal NCs, and over the years the reported NCs have revealed compelling NIR absorption and emission behavior [18, 19, 20, 21, 22, 23, 24, 25]. Nevertheless, NCs with high optical activity in the NIR‐II window (>1000 nm wavelength) are still scarce, with only a handful of examples reported to date [11, 26, 27, 28, 29].

Photothermal conversion has emerged as a vibrant research area over the last few years due to its significant application in medical fields, especially in photodynamic (PDT) and photothermal therapy (PTT) [6]. Although regular nanoparticles have long been used as PTT agents, they frequently suffer from poor efficiency and weak NIR absorption [30, 31, 32]. To mitigate these problems, new materials are critically needed. Atomically precise NCs have been found to be ideal materials that can overcome both issues due to their tunable NIR absorption and discrete energy levels; the latter facilitate electron‐vibration coupling for higher efficiency. While several NC systems have been investigated in this context [33, 34, 35, 36, 37, 38, 39, 40], systematic studies on NIR photothermy are still scarce. This is a notable gap, as NIR excitation affords deep tissue penetration, minimal scattering, and low autofluorescence; such properties that are critical for biological applications [41, 42]. Equally striking, the reported use of NC‐based photothermal platforms has focused almost exclusively on bioimaging, while strategies that harvest the generated thermal energy for alternative applications remain largely unexplored [43, 44, 45]. Since common photothermal agents are used in dilute solutions, the absorbed energy is rapidly dissipated into the solvent, whereas converting light into electricity requires a large, localized temperature rise in the solid state. Thermoelectric generators (TEGs) provide an efficient route for such thermoelectric conversion, if a voltage can be maintained between the hot and cold faces. Therefore, making a photothermal‐to‐electric device will require good photothermal conversion efficiency, high solid‐state temperature change, and stability under continuous irradiation, which APNCs can provide through their tunable NIR absorption and discrete energy levels that favor non‐radiative relaxation.

In this work, we report a systematic photothermal study on two alkynide‐protected alloy NCs: Au_24_Ag_20_(*^t^* BuPA)_24_Cl_2_ (*^t^* BuPA: 4‐*tert‐*butylphenylacetylene; henceforth referred to as M_44_) and Au_43_Ag_38_(*^t^* BuPA)_36_Cl_12_ (henceforth referred to as M_81_) in the solid state, organic solution, and aqueous phase. In solution, M_81_ and M_44_ NCs show remarkable photothermal conversion efficiency (PCE or η) of 91% and 69%, respectively, the highest values reported in the APNCs to date (Scheme 1). Using our previously developed polymer‐encapsulation strategy, both NCs can be phase‐transferred into an aqueous medium, opening a route to phototherapeutic applications. Remarkably, the solid state of the NCs under 808 nm irradiation exhibits a temperature increase of 150–226°C. To exploit this pronounced photothermal response, the NCs are integrated onto a Bi_2_Te_3_‐based thermoelectric generator (TEG), and an open‐circuit voltage of ∼125–150 mV is achieved under NIR laser irradiation. Overall, cascading NIR light into heat and subsequently into electricity establishes a conceptual blueprint for next‐generation devices that harness the otherwise under‐utilized NIR fraction of the solar spectrum, which is more than half of the total solar radiation.

**SCHEME 1:**
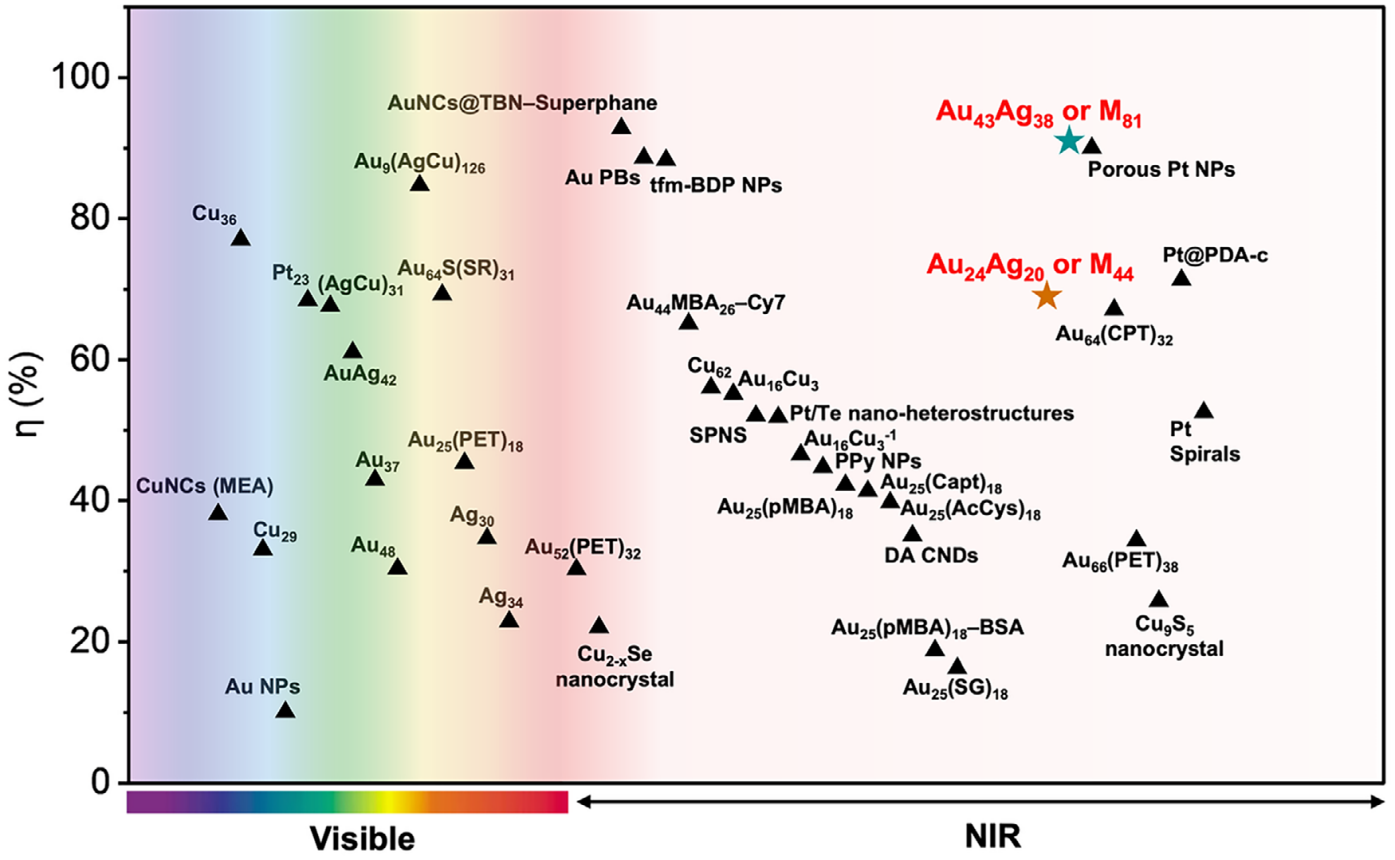
Selected nanostructured materials with various photothermal conversion efficiency values under visible to NIR laser irradiation. More details are provided in Table S1.

## 2 Results and Discussion

### 2.1 Synthesis and Purification

Both M_44_ and M_81_ NCs were synthesized by a modified, one‐pot procedure for preparing alkynide‐protected alloy NCs reported by Zhu and co‐workers [46]. In a typical synthesis (Figure S1), a mixture of chloro(dimethylsulfide)gold(I) (Me_2_SAuCl) and silver acetate (AgOAc) was dissolved in a mixed solvent of dichloromethane (DCM) and methanol (MeOH), followed by the addition of 4‐*tert*‐butylphenylacetylene (*^t^* BuPA) that had been briefly sonicated (5 min) with sodium methoxide (NaOMe). A reddish‐yellow Au–Ag–C≡CR polymeric complex intermediate was formed within minutes and was subsequently reduced by *tert*‐butylamine borane. The reaction was allowed to run for 24–72 h in the dark, followed by centrifugation to remove the by‐products (plasmonic nanoparticles). After thoroughly washing with MeOH multiple times, the size‐mixed NCs were extracted with DCM. In contrast to the earlier report that isolated M_44_ and M_81_ by tuning the reaction time and relying on direct crystallization [46], we employed thin‐layer chromatography (TLC) for post‐synthetic isolation/purification (Figure S2a), in which two distinct bands corresponding to M_44_ and M_81_ were observed; each was re‐purified and unambiguously identified by UV–vis–NIR absorption spectroscopy. The purified NCs were then used for further experiments. We note that the reaction time biases the product distribution. Specifically, quenching the reaction at ∼24 h favors the M_44_ formation, whereas extending to 36–72 h promotes the growth of M_81_. Furthermore, both M_44_ and M_81_ were suppressed and obtained as by‐products using our previous ripening‐based strategy for synthesizing Au_20_Ag_32_ NCs [27] (Figure S2b).

### 2.2 Structure and Optical Properties of M_44_ and M_81_

The M_44_ and M_81_ NCs are structurally related to each other by a monomer–dimer relationship. The monomeric M_44_ (Au_24_Ag_20_) is comprised of a hollow icosahedral Au_12_ kernel enclosed by a fullerene‐like Ag_20_ inner shell, then surrounded by 12 exterior Au atoms (Figure 1a). The clear separation of Au and Ag environments likely contributes to the pronounced electron–phonon coupling revealed in the optical properties. The dimeric M_81_ (Au_43_Ag_38_) can be viewed as a fusion of both the core and the inner shell of the M_44_: two Au_12_ units merge via an extra Au atom to form an Au_25_ kernel, while two Ag_20_ shells merge and lose two Ag atoms to form a M_38_ inner shell (Figure S3), further wrapped by 18 Au atoms and surface‐protecting ligands (Figure 1b). This face‐fusion is distinct from the side‐on biicosahedral fusion in M_52_ (Au_20_Ag_32_) and Au_23_ NCs [27, 47]. Kohn–Sham MO analysis reveals M_44_ is superatomic (resembling Au_25_) [48], while M_81_ possesses molecular‐like character (resembling Au_38_ and our earlier reported M_52_, Figure S4) [49]. Together, these provide an understanding of how the electronic structure dictates photoluminescence vs. photothermal behavior within the same co‐synthesized family.

**FIGURE 1:**
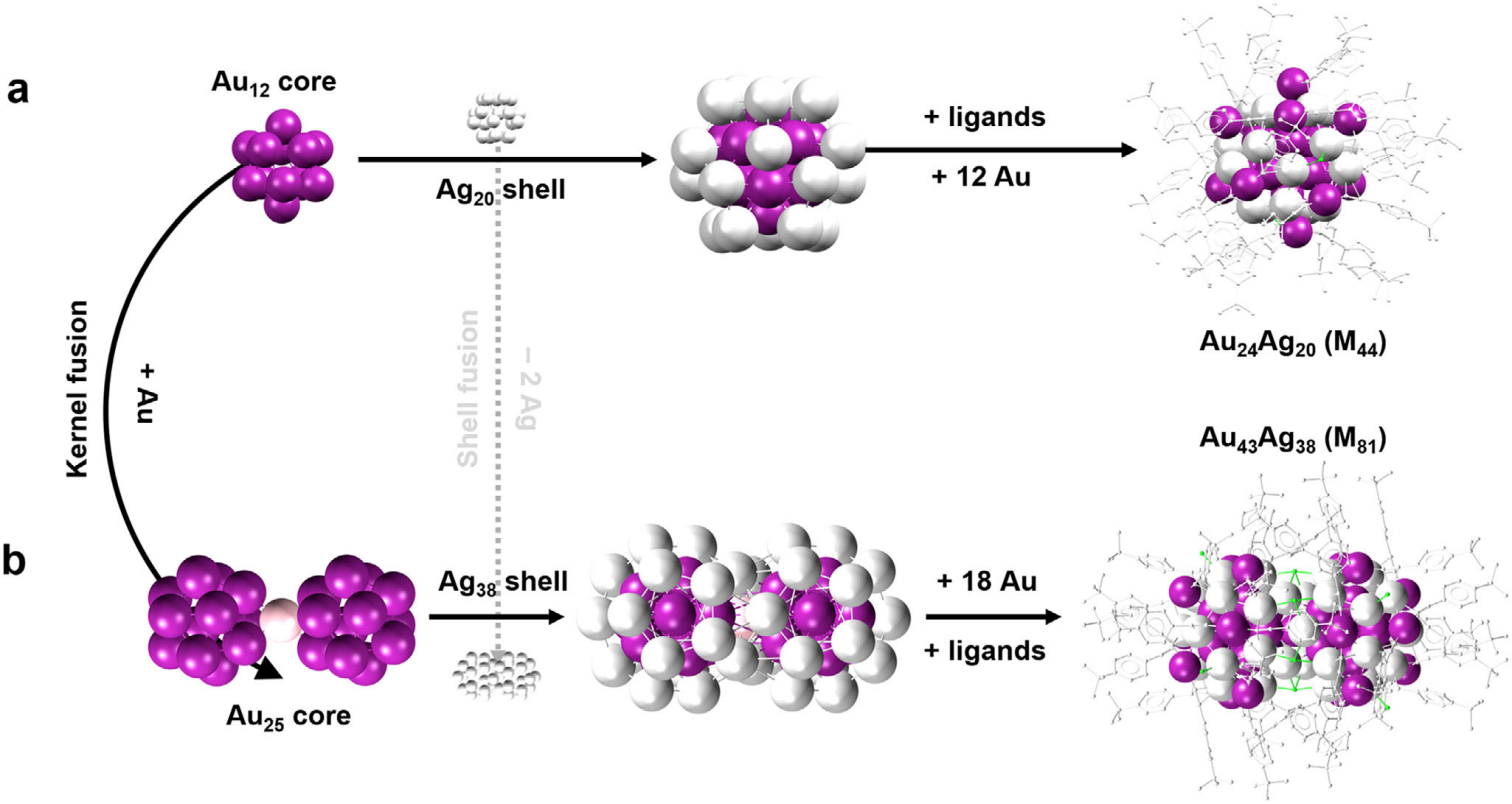
(a) Structural anatomy of M_44_ (Au_24_Ag_20_) NC: Au_12_ core surrounded by Ag_20_ shell, further capped by 12 Au atoms and ligands; (b) structural evolution of M_81_ (Au_43_Ag_38_) NC from M_44_: Au_25_ core, surrounded by Ag_38_ shell, further capped by 18 Au atoms and ligands. The Au_25_ core is formed by kernel fusion of two Au_12_ units and one Au atom addition, while the Ag_38_ shell is formed by two Ag_20_ shell fusion, sans 2 Ag atoms. Color code: purple = Au, gray = Ag, green = Cl, white = C; all H atoms are omitted.

The optical properties were investigated in deuterated chloroform (CDCl_3_, to avoid interference from vibrational overtone absorption of CHCl_3_) at room temperature. M_44_ shows absorption peaks at 365 nm, two convoluted peaks at 450 and 505 nm, and a broad band at 640 nm (Figure 2a), as well as tailing into the NIR (Figure S5a). M_81_ displays a peak at around 380 nm, two convoluted peaks at 410 and 495 nm (blue‐shifted relative to M_44_), and additional bands at 640 and 765 nm (Figure 2d), as well as tailing into the NIR‐II (Figure S5b). The monomer‐to‐dimer transition not only increases NIR absorption but also enhances the coefficient (ε) by ∼60 times (ε_640_ = 850 м^−1^ cm^−1^ vs. ε_765_ = 5 × 10^4^ м^−1^ cm^−1^, Figure S5c,d), consistent with the stronger transition dipole of the rod‐like M_81_.

**FIGURE 2:**
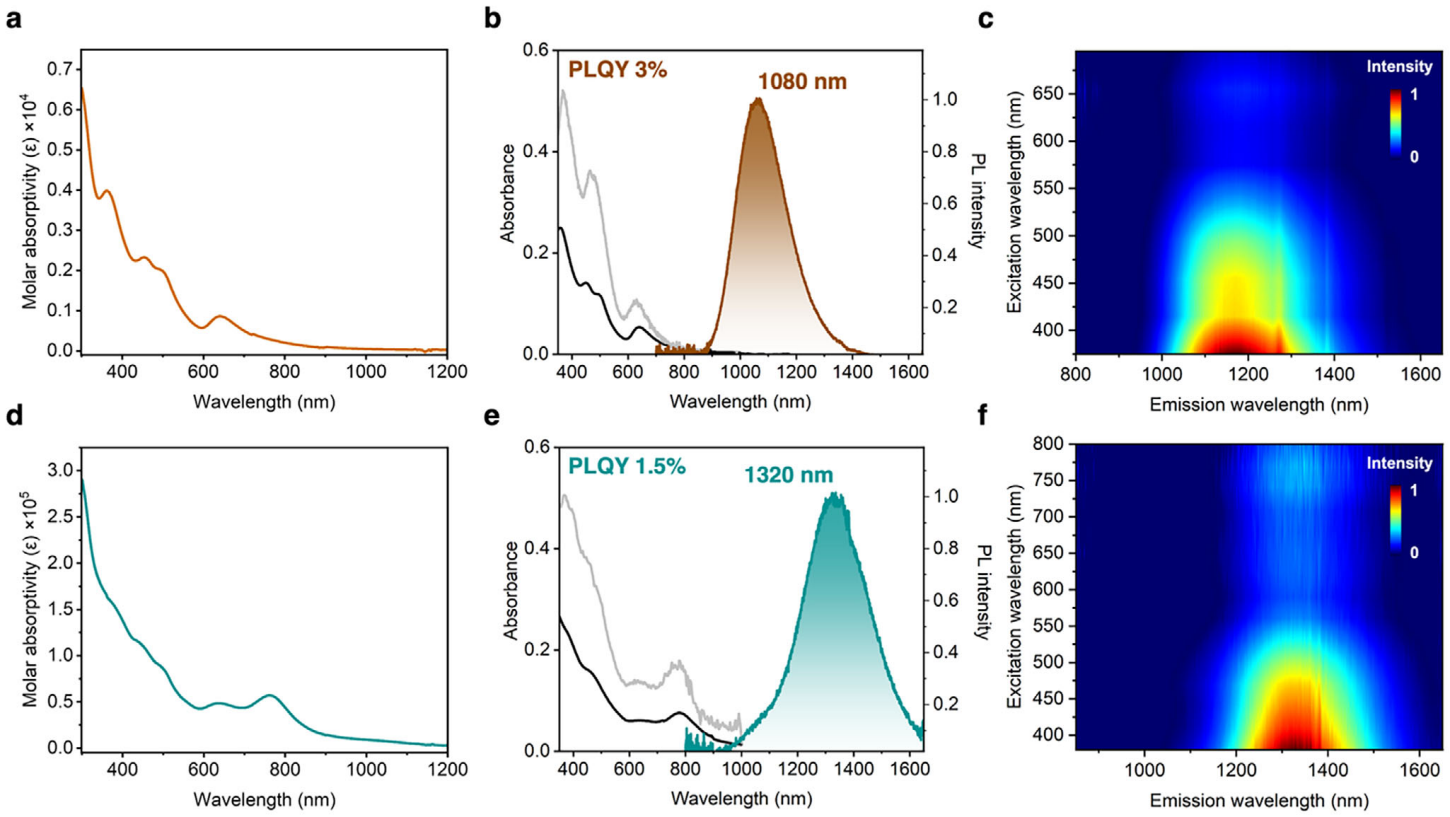
Optical properties of M_44_: (a) UV–vis‐NIR absorption spectrum, (b) comparison of absorption (black line) and PL excitation (gray line), and PL emission (brown shaded) spectra, (c) Excitation–emission contour map. Optical properties of M_81_: (d) UV–vis‐NIR absorption spectrum, (e) comparison of absorption (black line) and PL excitation (gray line), and PL emission (teal shaded) spectra, (f) Excitation–emission contour map.

Although these NCs were investigated for electrocatalysis previously [46, 50], their photophysical properties have remained unexplored. Herein, we evaluate the photoluminescence and photothermal functionality of these NCs. Both M_44_ and M_81_ exhibit NIR‐II photoluminescence (1000–1700 nm) peaking around 1080 and 1320 nm, respectively, upon 375 nm excitation (Figure 2b,e), with excitation‐independent profiles confirmed by excitation–emission maps (Figure 2c,f). PL decays for both NCs are biexponential: around 50 and 900 ns for M_44_, and 22 and 254 ns for M_81_ (Figure S6). To ascertain the presence of triplet emission states (indicated by the long lifetimes of hundreds of nanoseconds), we measured the PL spectra in He‐purged and O_2_‐purged CDCl_3_ medium. For the O_2_‐saturated solution of M_44_, the PL intensity decreases with the appearance of a sharp feature around 1275 nm compared to the He‐saturated solution; the 1275 nm signal is diagnostic of singlet‐oxygen phosphorescence (Figure S7) [51, 52]. However, M_81_, despite its long lifetime, does not exhibit the characteristic singlet‐oxygen peak when purged with O_2_. We attribute this to the fact that the emission energy of M_81_ (0.89 eV) is below the ^3^O_2_‐^1^O_2_ energy gap (0.97 eV); hence, not possible for triplet‐singlet energy transfer between phosphorescent M_81_’s triplet state and ^3^O_2_. Excited‐state dynamics probed by nanosecond and femtosecond transient absorption (fs‐TA and ns‐TA) show ground‐state bleach (GSB, blue) features consistent with the steady‐state absorption of both NCs (Figure S8). Global fitting analysis provides time constants of 0.97 ps and 36 ns (fs‐TA), 231 ns and 1.4 µs (ns‐TA) for M_44_, whereas M_81_ shows 1.4 ps and >7 ns (fs‐TA), 63 and 205 ns (ns‐TA). The ∼1 ps component for both NCs is attributed to internal conversion and intersystem crossing [53], and the longer ns components match well with the components obtained from time‐resolved emission lifetime measurements. The spectra of the long‐lived states in fs‐TA are also consistent with the ns‐TA data.

Except for the recently reported Au quantum rods (QRs) [54, 55], emission extending this deep into the NIR‐II, particularly in the case of M_81,_ is rare among atomically precise NCs. To date, only a handful of NCs (QRs of Au_78_, Au_96_ and Au_114_) have emission maxima at or beyond 1300 nm (Table S2) [11, 29]. Crucially, the excited‐state lifetime (∼300 ns) of M_81_ is considerably longer than those of QRs, offering an advantage for energy‐transfer and electron‐transfer applications, as well as NIR photocatalysis. The PL quantum yields (PLQYs), however, are quite low (3% for M_44_; 1.5% for M_81_ in CDCl_3_ solutions, measured relative to Au_42_ QRs, Figure S9), pointing to strong electron–phonon coupling that promotes non‐radiative relaxation, which contributes to the exceptional photothermal performance (described below). In the solid state, M_44_ PLQY reaches 6% in a polymethylmethacrylate (PMMA) matrix, while M_81_ shows a slight decrease (Figure S10). For biological applications, particularly in vitro and in vivo studies, aqueous solubility is essential. Following a polymer‐capping strategy recently introduced by Liu et al. [56], both M_44_ and M_81_ are successfully phase‐transferred into water by adding the FDA‐approved amphiphilic poloxamer Pluronic F‐127 to the organic solutions of the NCs, with a single or a few NCs in an NC@F‐127 supraparticle. The DLS spectra of both M_44_@F‐127 and M_81_@F‐127 show the size distribution of the supraparticles (Figure S11), peaking around 150 nm. In the supraparticle phase, the PLQY of M_44_ increases to 5%, while M_81_ drops to 0.4% (Figure S12). This divergent behaviour is consistent with recent reports which indicate that F‐127 encapsulation enhances the PLQY of spherical NCs while diminishing that of anisotropic (rod‐shaped) NCs [56]. The NCs are remarkably stable in ambient conditions, even after months (Figure S13), which benefits multi‐cycling in applications.

### 2.3 Photothermal Conversion

NIR photothermal conversion has recently found many applications, from renewable solar harvesting to advanced cancer phototherapy. Relative to UV and visible light, the NIR range offers distinct advantages for biomedical applications owing to reduced tissue scattering of NIR light (affording penetration depths of 5–20 mm), minimal light absorption by endogenous chromophores, and negligible autofluorescence [57, 58], which altogether enable deep‐tissue penetration with low phototoxicity. These render NIR‐photothermal therapy particularly attractive for PTT and PDT applications. Among the reported materials, including plasmonic nanoparticles, carbon nanomaterials, and conjugated organic polymers, atomically precise NCs have recently emerged as a new class of NIR‐active photothermal agents [59, 60, 61]. Unlike plasmonic nanoparticles, NCs convert absorbed NIR photon energy into localized heat through discrete electronic excitations, internal conversion, and electron‐phonon coupling pathways, with recent research demonstrating that various NCs show notably high photothermal conversion efficiencies [42, 62]. Importantly, recent in vivo work on Au_25_ and Au_44_ NCs has further demonstrated excellent biocompatibility and renal clearance [26, 32], underscoring NCs as prominent next‐generation photothermal agents [39, 63, 64]. Crucially, while NCs‐based photothermy has so far been developed for biomedical applications almost exclusively, the underlying heat generation as an energy resource has not yet been realized with APNCs.

Since M_44_ and M_81_ show low PLQYs, the non‐radiative relaxation from their excited states is predominant, which is driven by strong electron–phonon coupling, releasing the absorbed energy as heat primarily. Thus, we systematically investigated the photothermal conversion performance of M_44_ and M_81_ in multiple forms, including organic solution, solid state, and aqueous medium. For all the experiments, we used a setup comprising an industry‐standard 808 nm laser (using various optical power densities (OPDs) from 0.1 to 2.5 W·cm^−2^) for irradiation and a thermal camera for real‐time temperature monitoring. In organic solution, in accordance with common literature practices, we used toluene as the solvent owing to its high boiling point and the long‐term stability of the NCs in this medium. It is worth noting that, although recently there has been a surge in testing various NCs for photothermal applications, systematic photothermal measurements involving variable concentrations, optical power density, and sample absorbance are still scarce. Through our systematic studies, we have tried to disentangle an ordered relationship between maximum temperature rise (ΔT_max_) and photothermal conversion efficiency (η): two parameters that are critical for different applications.

To our excitement, M_81_ shows a record‐high η of 91% when irradiated with 0.5 W·cm^−2^ at an absorbance of 0.2 optical density (OD) at 808 nm, with a maximum temperature rise (ΔT_max_) of 11.2°C. Upon increasing the OPD from 0.5 to 2.5 W·cm^−2^, η drops to 38%, while ΔT_max_ gradually increases to as high as 35.4°C (T_max_ = 59.4°C) (Figure 3). The concentration‐dependent measurements at absorbances of 0.2, 0.5, and 1.0 OD (at 808 nm) reveal a clear inverse relationship between η and ΔT_max_, in line with previous reports [34, 38]. Interestingly, ΔT_max_ reaches as high as 71.6°C (T_max_ = 96.8°C) at 1 OD_808 nm_ and 2.5 W·cm^−2^. Likewise, the M_44_ NC also shows high efficiency and temperature change, with η_max_ of 69% and ΔT_max_ of 58°C; the corresponding OPD‐ and absorbance‐dependent trends are summarized in Figure 4, like M_81_. All the heating curves and the cooling time plots, which are required to calculate the efficiencies, are provided in Figures S14‐S17. Next, we tested whether the NIR‐II absorption tail (>1000 nm) of M_81_, which is not accessible by M_44_, could be utilized for photothermy at truly tissue‐transparent wavelengths of NIR‐II. Under 1064 nm excitation, M_81_ produced a ΔT_max_ of 9°C with a peak η of ∼10%, despite its very weak extinction in this window (Figure S18). While low in absolute values, this result is among the first demonstrations of direct NIR‐II photothermy from APNCs at NIR‐II wavelengths and constitutes an encouraging step toward the rational development of NIR‐II‐active NC photothermal agents. Taken together, the high η values and temperature rises across a broad range of conditions encourage us to evaluate the NCs for additional applications.

**FIGURE 3:**
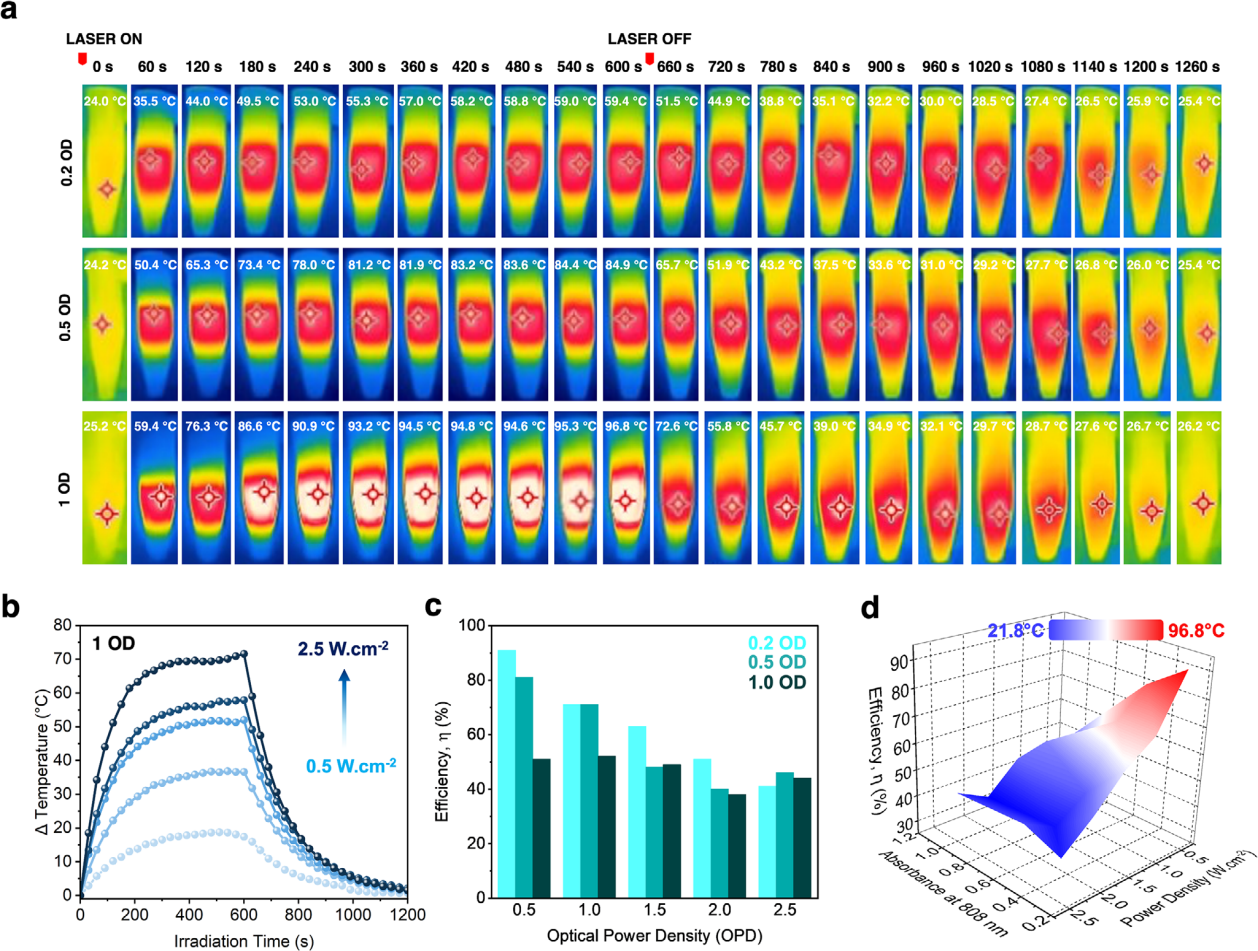
(a) Infrared thermal camera images of the ΔT_max_ for M_81_ NCs in toluene at various absorbances at 2.5 W·cm^−2^ OPD during photothermal conversion experiments using an 808 nm laser. For each frame, red to white is the highest temperature, while yellow to blue are the lowest temperature regions. (b) Heating curve of the M_44_ NCs with an absorbance of 1 OD at varying OPDs. (c) Bar graph representing the photothermal conversion efficiency, η (y‐axis), at different concentrations (shaded) and different OPDs (*x*‐axis). (d) 3D contour plot representing the relationship between the OPDs (*x*‐axis), absorbance at 808 nm (y‐axis), efficiency (z‐axis), and recorded solution temperature (shaded).

**FIGURE 4:**
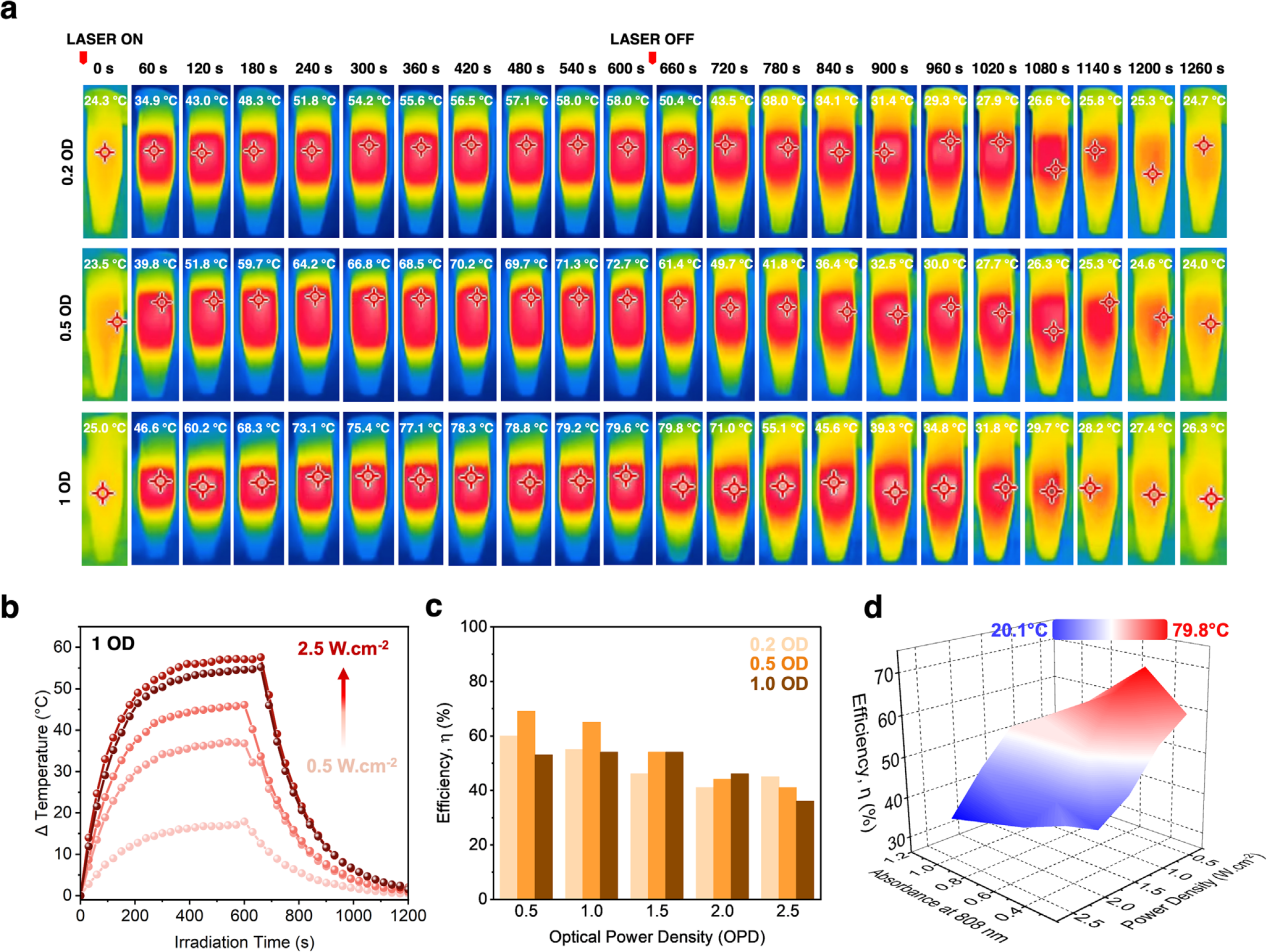
(a) Infrared thermal camera images of the ΔT_max_ for M_44_ NCs in toluene at various absorbances at 2.5 W·cm^−2^ OPD during photothermal conversion experiments using an 808 nm laser. For each frame: red to white is the highest temperature, while yellow to blue are the lowest temperature regions. (b) Heating curve of the M_44_ NCs with an absorbance of 1 OD at varying optical power densities. (c) Bar graph representing the photothermal conversion efficiency, η (y‐axis), at different concentrations (shaded) and different OPDs (*x*‐axis). (d) 3D contour plot representing the relationship between the OPDs (x‐axis), absorbance at 808 nm (y‐axis), efficiency (z‐axis), and recorded solution temperature (shaded).

As discussed in the optical properties section, both NCs were transformed into water‐soluble supraparticles via F‐127 encapsulation. Here, the aqueous samples are further evaluated for photothermal performance in water. Both M_44_@F‐127 and M_81_@F‐127 show η_max_ of 49% and 50% with ΔT_max_ of 20°C and 19°C, respectively (Figure 5). Efficiencies approaching 50% in water are particularly noteworthy given the high heat capacity of water, which slows the cooling kinetics used in η extraction (see SI, Figures S19‐S21). Moreover, the moderate, but not excessive ΔT_max_ values obtained here are well suited to photothermal therapy, which is sufficient to induce tumor cell ablation yet low enough to spare surrounding healthy tissue (otherwise affected at higher hyperthermic temperatures). Both M_44_@F‐127 and M_81_@F‐127 supraparticles therefore are promising for biological PTT applications. However, in the future, more studies are still needed to understand their biocompatibility, stability, and biosafety under physiological conditions in order to demonstrate their therapeutic effectiveness.

**FIGURE 5:**
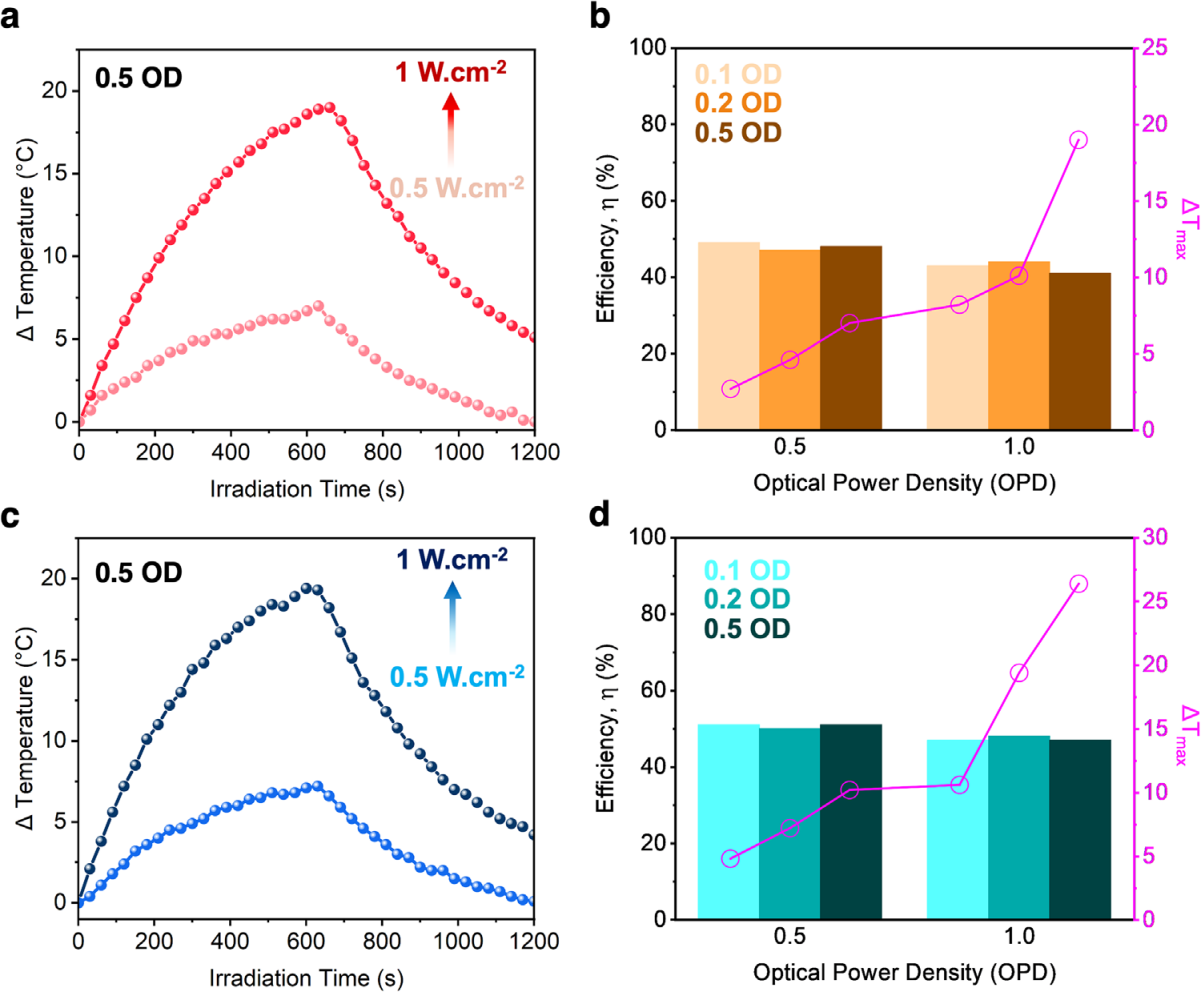
(a) Heating curve of the M_44_ NC@F‐127 supraparticles with absorbance of 0.5 OD at varying OPDs. (b) Bar graph representing the photothermal conversion efficiency η (y‐axis) at different concentrations (brown shaded) and different OPDs (black *x*‐axis), and their corresponding ΔT_max_ (pink *y*‐axis). (c) Heating curve of the M_81_ NC@F‐127 supraparticles with an absorbance of 0.5 OD at varying OPDs. (d) Bar graph representing the photothermal conversion efficiency η (y‐axis) at different concentrations (teal shaded), and different OPDs (black x‐axis), and their corresponding ΔT_max_ (pink *y*‐axis).

Next, we turn to the photothermal conversion experiments in the solid state. For solid‐state applications, to emulate photonic devices, we used thin films of M_44_ and M_81_ NCs embedded in a polymethyl methacrylate (PMMA) matrix and irradiated such films under identical conditions. Surprisingly, the M_44_ and M_81_ NCs reach a ΔT_max_ of 226°C and 155°C, respectively, when excited under 2.5 W·cm^−2^ OPD (Figure S22). This high photothermal heat generation capability should be useful in the future for device fabrication, provided the NCs are encapsulated in a stable heat‐resistant polymer.

Stability is a critical consideration in such experiments, as materials exhibiting large ΔT can readily undergo self‐induced thermal degradation. The UV–vis–NIR spectra were recorded before and after irradiation in all three media (Figures S23–S25); no discernible spectral change was observed, verifying the robust stability of both NCs.

These results highlight a frequently overlooked point in the photothermal literature: η and ΔT_max_ are counteracting, and optimizing for one generally sacrifices the other. These NCs thus can be used for dual purposes: moderate ΔT with high η for photothermal therapy on one hand, and large ΔT for NIR‐driven energy‐harvesting devices on the other. Taken together, the combination of strong heat generation, record‐high photothermal conversion efficiency amongst atomically precise nanomaterials, and exceptional stability motivates us to explore the downstream conversion of NIR‐driven heat into electrical energy (see next section).

### 2.4 Thermoelectric Conversion

The combination of record photothermal efficiency and solid‐state ΔT_max_ exceeding 200°C places these NCs in a regime where the generated heat is not merely a byproduct of photoexcitation, but a usable energy “currency”. We therefore tested whether the NIR‐driven thermal output of the film could be coupled to a thermoelectric device to produce electricity, thereby enabling a cascade conversion of light‐to‐heat‐to‐electricity. Currently, solar energy harvesting is limited predominantly to the UV and visible regions, despite the fact that more than half of solar energy lies in the NIR window. The ability to harness this abundant and underutilized NIR portion of the solar spectrum and transduce it sequentially into thermal and then electrical energy represents an exciting route toward sustainable energy conversion. Thermoelectric generators (TEGs), which convert thermal energy directly into electrical output via the Seebeck effect, are a perfect device to capture the heat generated by photothermal nanomaterials under NIR irradiation [65, 66, 67]. By fabricating an NIR‐active metal NCs‐TEG interface, we demonstrate a photothermally driven electrical current. To the best of our knowledge, this is the first TEG application of atomically precise NCs.

To generate electricity in TEGs, a temperature difference or gradient needs to be maintained across two interfaces, namely a hot side and a cold side. This temperature gradient drives the directional charge‐carrier diffusion through the thermoelectric material, generating a measurable open‐circuit voltage. The greater the thermal gradient between the two sides, the larger the resulting open‐circuit voltage and electrical current output. In our case, we deposited a layer of the NCs on the top of the hot side of a commercial Bi_2_Te_3_ TEG, while keeping the cold side intact. While efficient TEG operation typically employs an active heat sink or ice bath on the cold side, we intentionally omitted any such external cooling in order to demonstrate electricity generation under minimal, device‐integrated conditions. A schematic diagram of our fabricated device is shown in Figure 6a. In our experiments, we used the same 808 nm laser to shine on the NCs film on the TEG device and measured voltage and current using an electrochemistry setup. With the M_44_ and M_81_ NCs, we observed a constant open‐circuit voltage of 146 and 127 mV sustained over 900 s of continuous irradiation, respectively (Figure 6b,c). We note that, for TEG and other applications, not only the PCE values but also the absorption coefficients are important. Since the molar absorptivity of M_81_ is one order higher than M_44_, much more M_44_ loading on the TEG device is required for the same absorption. While higher PCE does not guarantee higher ΔT_max_, the amount of NCs is generally proportional to ΔT_max_; thus, the M_44_ NC shows a much higher ΔT_max_ in the solid state (Figure S21) as well as in TEG.

**FIGURE 6:**
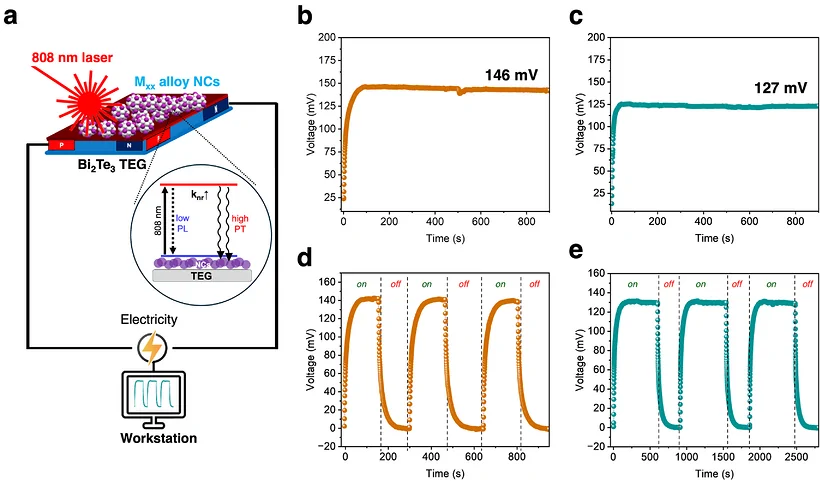
(a) Schematic diagram of the fabricated photo‐thermo‐electricity cascade device using the NCs@TEG. Open‐circuit voltage generated through (b) the M_44_/TEG device (orange) and (c) the M_81_@TEG device (teal). Recyclability experiments (3 cycles) for (d) the M_44_/TEG device and (e) the M_81_@TEG device.

Cycling experiments showed that both NC/TEG devices can be repeatedly switched on and off over multiple cycles without any measurable degradation in output voltage (Figure 6d,e), hence, the robustness of the NCs on the device, which is important for practical implementation. Control experiments on bare TEG in the absence of NCs produced no detectable voltage, consistent with the negligible NIR absorption of Bi_2_Te_3_ (Figure S26). Introducing a commercial thermal paste at the NC‐TEG interface to facilitate heat transfer yielded comparable voltages, indicating that direct NC deposition already provides efficient thermal coupling to Bi_2_Te_3_. Finally, we emphasize that the present experiments probe only a narrow spectral slice (around 808 nm) of the NCs' optical absorption, whereas both M_44_ and M_81_ exhibit broad absorption spanning the visible to the NIR. Their absorption profiles overlap well with the solar spectrum (Figure S27), suggesting that direct sunlight could, in principle, drive such photo–thermo–electric cascades. By encapsulating NCs within high temperature stable carbon or SiO_2_ shell [68, 69], the NC@TEG combination can potentially operate at higher temperatures under focused solar light. We anticipate that our conceptual idea may motivate the design of NC‐based next‐generation devices capable of harvesting the unutilized NIR portion of the solar spectrum through sequential light‐to‐heat‐to‐electricity conversion. More broadly, this work introduces quantum‐sized NCs as components in functional devices, expanding the applications of NCs as solution‐phase “chromophores”, and suggests that the electron–phonon coupling typically regarded as just an “unwanted” (i.e., luminescence quenching) can instead be leveraged as a design principle for near‐infrared photonic energy harvesting.

## 3 Conclusion

In summary, we have established atomically precise bimetallic nanoclusters as a new class of NIR‐driven photothermoelectric transducers, demonstrating a functional light‐to‐heat‐to‐electricity cascade device enabled by record photothermal conversion efficiencies and unprecedented solid‐state temperature rise. We have systematically investigated their photophysical and photothermal behavior. Both NCs exhibit NIR‐to‐SWIR photoluminescence, with emission maxima at 1080 nm (M_44_) and 1320 nm (M_81_)–rare examples of APNCs emitting so far into the infrared. Complementary studies established several routes for enhancing the PLQY and phase‐transfer into aqueous media via Pluronic F‐127 encapsulation, affording water‐soluble supraparticles suitable for biological applications. Most strikingly, the NCs display outstanding photothermal conversion, with solution‐phase efficiencies of 69% (M_44_) and a record near‐unity value of 91% (M_81_)–the highest reported for any APNC to date, in solution, as far as our knowledge. In the solid state, the same APNCs sustain an exceptionally large temperature change, with ΔT_max_ exceeding 200°C, all while remaining stable. Capitalizing on this combination of high efficiency, large ΔT, and remarkable stability, we fabricated NC/Bi_2_Te_3_ TEG devices that convert NIR light into electricity through a Seebeck‐effect‐mediated photo–thermo–electric cascade, generating stable voltages of 146 mV (M_44_) and 127 mV (M_81_) with excellent recyclability and reproducibility. Nonetheless, key bottlenecks include maximizing power delivery through thinner media, broadening the NIR–SWIR absorption for solar applications, better thermal coupling, and long‐term usability. Linking the atomic structure to electron–phonon coupling will further enable rational design and better photothermally efficient NCs. We anticipate that this work will stimulate further efforts in the rational design of NIR‐active photothermal NCs and will open new avenues for their utilization in both phototherapy and NIR‐driven solar energy harvesting devices.

## Author Contributions

**Avirup Sardar**: investigation, Writing – original draft. **Abhrojyoti Mazumder**: investigation. **Weijie Ji**: investigation. **Guiying He**: investigation. **Yitong Wang**: investigation. **Lianshun Luo**: investigation. **Sihan Chen**: investigation. **Rongchao Jin**: writing – review and editing, project administration.

## Funding

This work was supported by the NSF (CHE‐2404213).

## Conflicts of Interest

The authors declare no conflicts of interest.

## Supporting information




**Supporting File**: advs78061‐sup‐0001‐SuppMat.pdf.

## Data Availability

The data that support the findings of this study are available on request from the corresponding author. The data are not publicly available due to privacy or ethical restrictions.
