# Supplementary material for: Near‐Unity Photothermal Conversion in Bimetallic M44 and M81 Nanoclusters Enables NIR‐Driven Photo‐Thermo‐Electricity Generation
Source: Adv Sci (Weinh). 2026 Sep 30:e78061. Online ahead of print. doi: 10.1002/advs.78061 (PMC13626284; doi:10.1002/advs.78061)
Supplement: Supplementary file 1 [file ADVS-9999-e78061-s001.pdf]

Supporting Information

Near-Unity Photothermal Conversion in Bimetallic M_{44} and M_{81} Nanoclusters Enables NIR-Driven Photo-Thermo-Electricity Generation

Avirup Sardar,[§] *Abhrojyoti Mazumder,*[§] *Weijie Ji, Guiying He, Yitong Wang, Lianshun Luo, Sihan Chen, and Rongchao Jin**

Department of Chemistry, Carnegie Mellon University, Pittsburgh, Pennsylvania 15213, United States

*Corresponding author, email: rongchao@andrew.cmu.edu, rbj5326@psu.edu

[§] The authors have contributed equally to this work

S1. EXPERIMENTAL

S1.1. Chemicals

Tetrachloroauric (III) acid ($HAuCl_4 \cdot 3H_2O$, 99.999% metal basis, Sigma-Aldrich), dimethyl sulfide (SMe_2, >99%, Sigma-Aldrich), 4-*tert*-butylphenylacetylene ($^tBuPhC{\equiv}CH$, 96%, Thermo Scientific Chemicals), silver acetate ($AgCH_3COO$, 98%, Sigma-Aldrich), sodium methoxide (NaOMe, for synthesis, Sigma-Aldrich), *tert*-butylamine borane ($(CH_3)_3CNH_2 \cdot BH_3$, powder, 97%, Sigma-Aldrich). Solvents: ethanol, ethyl ether, toluene, methanol, dichloromethane (DCM), *n*-hexane, chloroform, acetone, 2-methyl tetrahydrofuran, chloroform-d and toluene-d_8 (HPLC grade for all solvents). All chemicals were used as received without further purification. Thin-layer chromatography (TLC) plates were purchased from iChromatography (silica gel, 250 μm).

S1.2. Synthesis Procedures

Synthesis of chloro(dimethylsulfide)gold(I) ($AuCl(SMe_2)$)

$HAuCl_4 \cdot 3H_2O$ (500 mg, 1.27 mmol) was dissolved in ethanol (20 mL), followed by the addition of SMe_2 (280 μL, 3.81 mmol), and the solution was vigorously stirred for 2 hours. After stirring, the white precipitate was collected by centrifugation, which was washed with ethyl ether and finally dried to give the product as a white powder

Synthesis of $Au_{24}Ag_{20}(C_{12}H_{13})_{24}Cl_2$ (M_{44}) and $Au_{43}Ag_{38}(C_{12}H_{13})_{36}Cl_{12}$ (M_{81})

$Au_{24}Ag_{20}$ (M_{44}) and $Au_{43}Ag_{38}$ NCs (M_{81}) were synthesized with modifications to a method reported before. In a typical synthesis a 50 mL round-bottom flask was charged with 58.8 mg (0.2 mmol) chloro(dimethylsulfide)gold(I) [($AuCl(SMe_2)$)] and 33.4 mg silver acetate (AgOAc) dissolved in 15 mL DCM/methanol (2:1). The colorless solution was allowed to stir for a few minutes. In a small vial 20 μL 4-*tert*-butylphenylacetylene (^{t}BuPA) was sonicated in the presence of 10mg sodium methoxide (NaOMe). After 5 minutes of sonication, the ^{t}BuPA solution was added to the original solution under vigorous stirring forming an orange-red solution. After 20 minutes 20 mg of borane *tert*-butylamine [$(CH_3)_3CNH_2 \cdot BH_3$] was added. Within a few seconds, the solution turned black, and the reaction was allowed to proceed in the dark for 24-72 hours. Stopping the reaction early results in higher yield of $Au_{24}Ag_{20}$ while continuing for longer times favors formation of $Au_{43}Ag_{38}$ NCs. After the reaction was stopped, the solvent was removed via rotary evaporation, and the crude product was washed with methanol multiple times, extracted using dichloromethane and concentrated for TLC separation. The mixture of NCs was pipetted onto a TLC plate, and the separation was conducted in a developing tank (developing solvent 1:3 (v/v) DCM:*n*-hexane). The broad brown band ($Au_{24}Ag_{20}$), or the bottom dark green band ($Au_{43}Ag_{38}$) was cut off, and one further round of TLC was performed for enhanced purity. The as-obtained bands were dissolved in DCM for initial characterization. The yield was ~25% for $Au_{24}Ag_{20}$ and ~10% $Au_{43}Ag_{38}$ (Au atom basis).

S1.3. Characterization

Steady-State UV-Vis-NIR Measurements

The UV–Vis–NIR spectra of the NC solutions were measured on a UV-3600 Plus spectrophotometer (Shimadzu) with a wavelength range of 185-3300 nm.

Steady-state Photoluminescence Measurements

Steady-state photoluminescence spectra were measured using an Edinburgh FLS-1000 spectrofluorometer. Near-infrared PL was measured using a broadband InGaAs-based PMT-1700 detector (wavelength range: 500-1650 nm) cooled down to -80 °C by liquid nitrogen. For the extended NIR-II to SWIR region of M_{81} PL spectra, a liquid nitrogen cooled InAs photovoltaic detector (wavelength up to 3100 nm) with lock-in amplification was used; the excitation and emission slit bandwidths were set to 15 and 40 nm, respectively, and a dwell time of 0.2 s for 10 runs.

Time-Resolved Photoluminescence Measurements

Time-correlated measurements were performed using an EPL-450 picosecond pulsed diode laser (Edinburgh Instruments) in multi-channel scaling (MCS) mode. The excitation wavelength from EPL-450 was 450 nm with a variance smaller than 5 nm. The pulse frequency was varied in the range of 100 kHz – 25 kHz, and the detection wavelength was set at 1100 nm and 1320 nm for M_{44} and M_{81} NCs, respectively.

Relative Quantum Yield Determination

The relative quantum yield (Φ_S) of the sample is calculated by using:

$$\phi_S = \phi_R \left(\frac{I_S}{I_R}\right)\left(\frac{1-10^{-A_R}}{1-10^{-A_S}}\right)\left(\frac{n_S}{n_R}\right)^2$$

In this context, ϕ_R denotes the quantum yield of the reference (standard), *I* represent the integrated photoluminescence (PL) intensity, *A* corresponds to the absorbance of the solution at the excitation wavelength, **n** stands for the refractive index of the solvent, and the subscripts (*S* and *R*) distinguish between the sample and the reference, respectively.

Transient Absorption Spectroscopy

Transient absorption measurements were performed using a broadband pump-probe setup, which is pumped by a 1 kHz Ti:Sapphire laser system (Spitfire, Spectra-Physics). The pump wavelengths (365 nm) were generated by a commercial optical parametric amplifier (TOPAS-C, Light Conversion). For femtosecond TA measurements, the supercontinuum probe light is generated by focusing the fundamental pulse (800 nm) into a sapphire plate. The probe light is split into signal and reference beams. The pump-probe delay was controlled by a mechanical delay line. For nanosecond TA measurements, the probe light is generated with a fiber laser (Leukos), and the delay times up to microseconds are controlled by an electronic delay configuration. TA measurements were performed in toluene, and the optical density of solution was adjusted to ~0.2 OD (2 mm cuvette) at excitation wavelength. The polarization of pump and probe pulse was set to magic angle (54.7°) to measure the isotropy signal. The transient absorption spectra were further analyzed using the publicly available program Glotaran based on the statistical fitting package TIMP (*J. Stat. Soft.* **2012**, *49*, 1–22 ; *Biochim. Biophys. Acta - Bioenerg.* **2004**, *1657*, 82–104).

Photothermal Conversion Experiments

To evaluate the photothermal conversion of the NCs, a home-built setup was used consisting of an 808 nm or 1064 nm laser with variable aperture for NC irradiation, HSFTools HP96 Thermal Camera for temperature tracking.

The photothermal performance of the NC samples was evaluated by monitoring the temperature increase under continuous laser irradiation. For both organic and aqueous phases generally 1.5 mL NC solutions of desired concentrations were taken and transferred into a 2 mL Eppendorf tube. A continuous wave 808 nm or 1064 nm near-infrared laser was directed onto the sample. The laser power density was adjusted using neutral density filters and measured prior to each experiment with a SCIENTECH Astral AD30 power meter. During the measurement, the laser beam was focused onto the center of the Eppendorf to ensure uniform illumination of the sample volume. The temperature change of the sample was recorded as a function of irradiation time using an infrared thermal camera positioned in one side of the Eppendorf. The samples were irradiated for 10 min, until the temperature reached a steady state, followed by a natural cooling period after the laser was turned off. Control experiments were carried out under identical conditions using the pure solvent to distinguish the intrinsic photothermal effect of the sample from background heating.

For quantitative analysis, the photothermal conversion efficiency can be calculated from the heating and cooling curves using the standard energy balance method, which accounts for the laser input energy, heat dissipation to the surroundings, and solvent background contribution.

The photothermal conversion efficiency (η) equation used is as follows:[1–3]

$$\eta = \frac{hS\Delta T_{max}}{I\,(1 - 10^{-A_\lambda})}$$

(I =laser power density, A_λ = absorbance of NCs at the irradiated wavelength, h = heat transfer coefficient, S = total surface area of the vial, ΔT_{max} = maximum temperature change)

For photothermal conversion efficiency in water a modified equation was used:[4]

$$\eta = \frac{hS\Delta T_{max} - q_{dis}}{I\,(1 - 10^{-A_\lambda})}$$

where q_{dis} accounts for the heat dissipated from blank water under same illumination.

To find out the hS, we replace it with the the heat time constant, τ_s . The relation between hS and τ_s is given by:

$$\tau_s = \frac{\sum m_i C_{p,i}}{hS}$$

(m_i and $C_{p,i}$ are the mass and heating capacity of the systems respectively. The mass of the NCs were too negligible in compared to the NCs, so the values for the solvent toluene, or water was taken. For toluene: m_i = 1.3 g, $C_{p,i}$= 1.71 J/(g °C), for water: m_i = 1.5 g, $C_{p,i}$= 4.18 J/(g °C))

During the cooling time (when the laser is off), we can find out the τ_s by introducing a dimensionless constant θ, which is basically:

$$\theta = \frac{T - T_{surr}}{T_{max} - T_{surr}}$$

(T = temperature of system, T_{max} = maximum temperature reached, T_{surr} = initial temperature)

$$t = -\tau_s ln\theta$$

τ_s is calculated from the slope of cooling time (t) *vs* –ln θ plot and plugging in the values in the position of hS.

Photo-Thermo-Electric Device Measurements

The photothermoelectric device was fabricated by depositing a NC layer over a commercial TEG device purchased from TECTEG (Model No. TEG1-1263-4.3). Highly concentrated NC solutions in DCM were first made and then deposited evenly over the TEG surface. Typically, 2 mL of toluene solution of 1 OD_{808nm} NCs was prepared. Then, using a glass pipette, the NC solution was drop-casted onto the Hot side of the TEG plate. Special care was taken to make sure the whole side was covered, with limited overlapping drops of the NCs. After that, the NC@TEG device was allowed to dry slowly. After complete solvent evaporation for 2-3 hours, the NC@TEG device is ready for TEG testing. The voltage across the hot and cold side of the PTEG device was measured using CHI 620C electrochemical workstation.

S2. SUPPORTING FIGURES

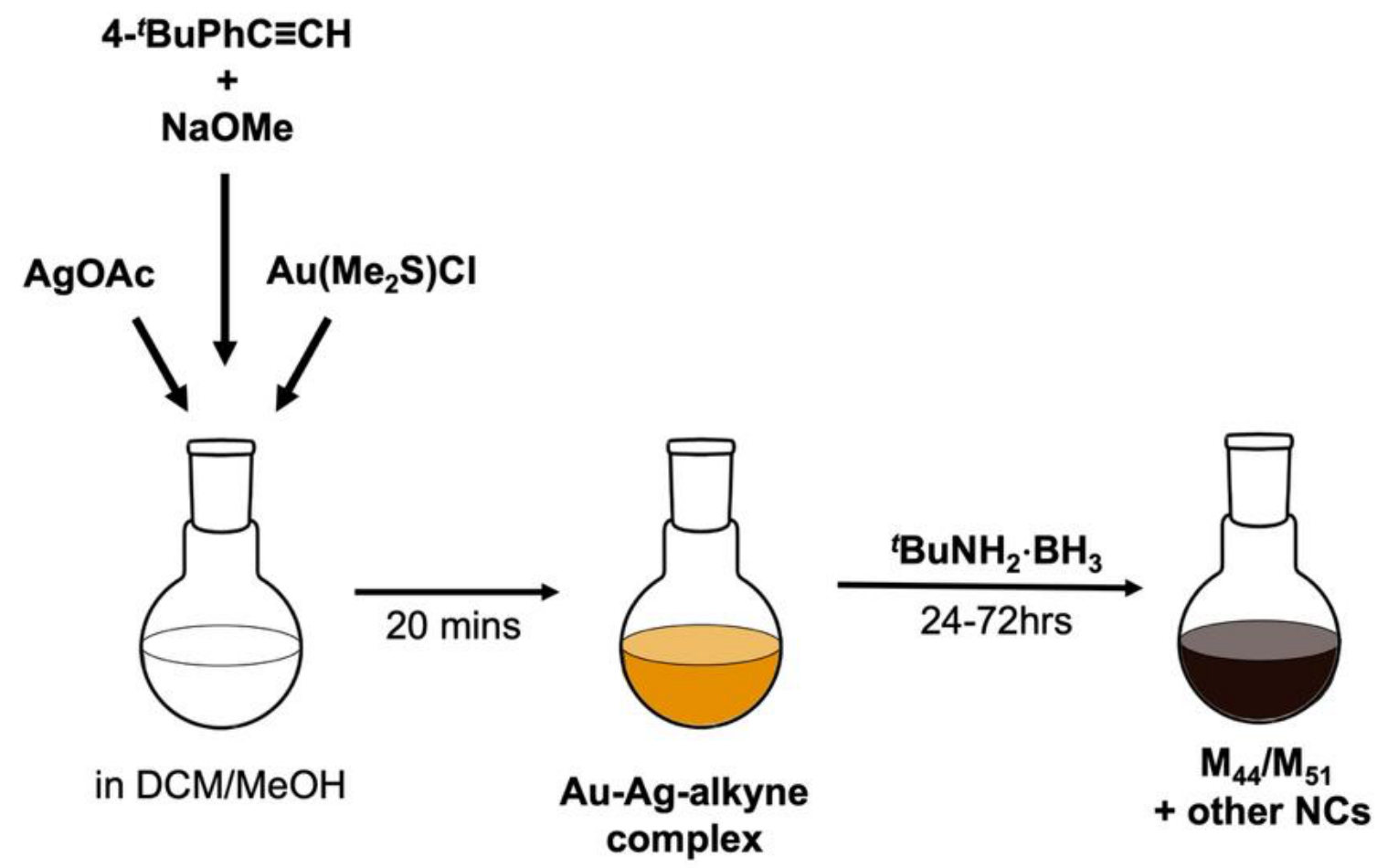

Figure S1. Synthetic route for the preparation of M_{44} and M_{81} NCs.

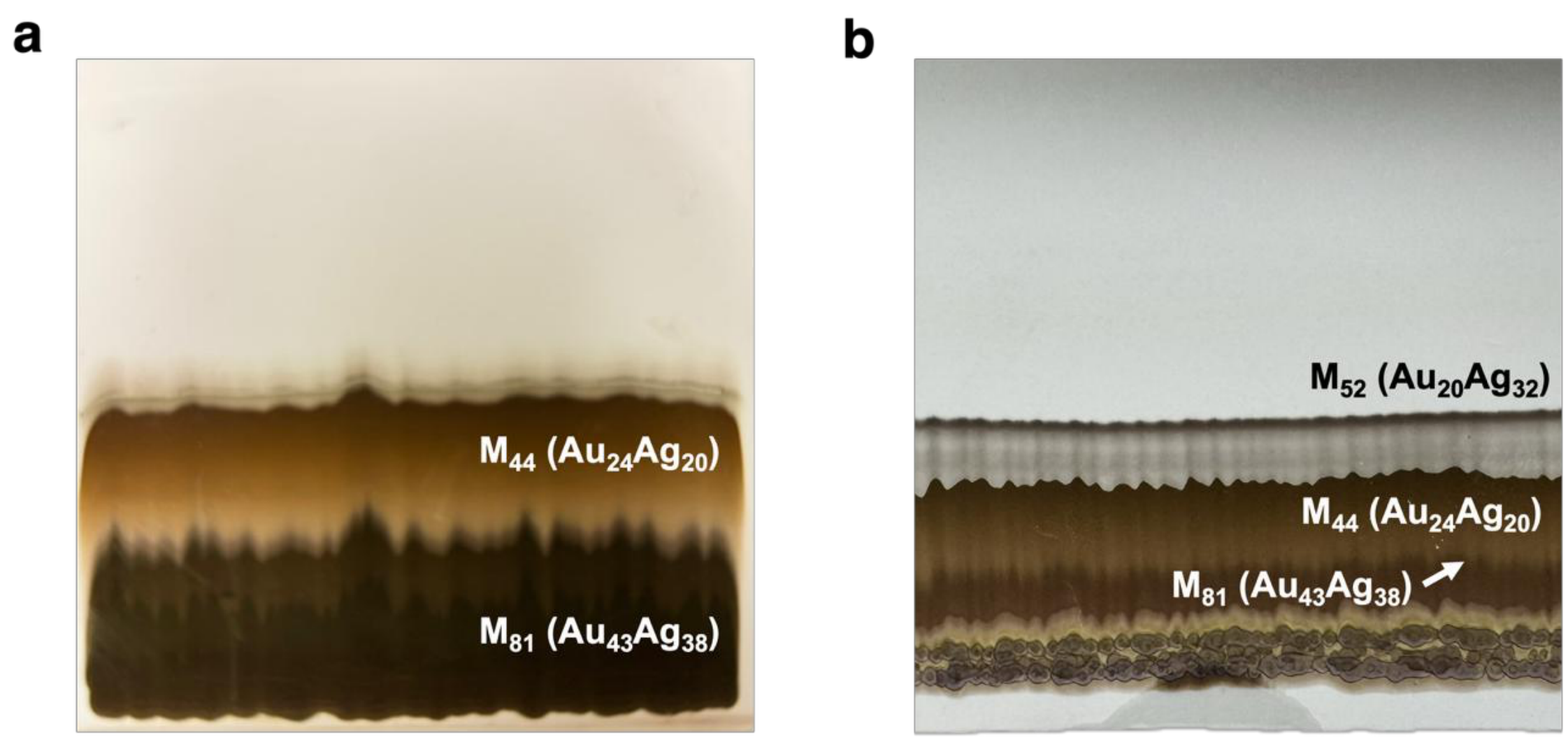

Figure S2. (a) TLC separation of M_{44} and M_{81} from the product mixture made by the procedure in Fig S1, with the top brown band and the bottom greenish-black band being M_{44} and M_{81}, respectively). (b) TLC separation of M_{44}, M_{81} and M_{52} from the product mixture made using the process in ref [5]; M_{81} was obtained in trace amounts *via* this process, marked by the white arrow to the thin faint green band.

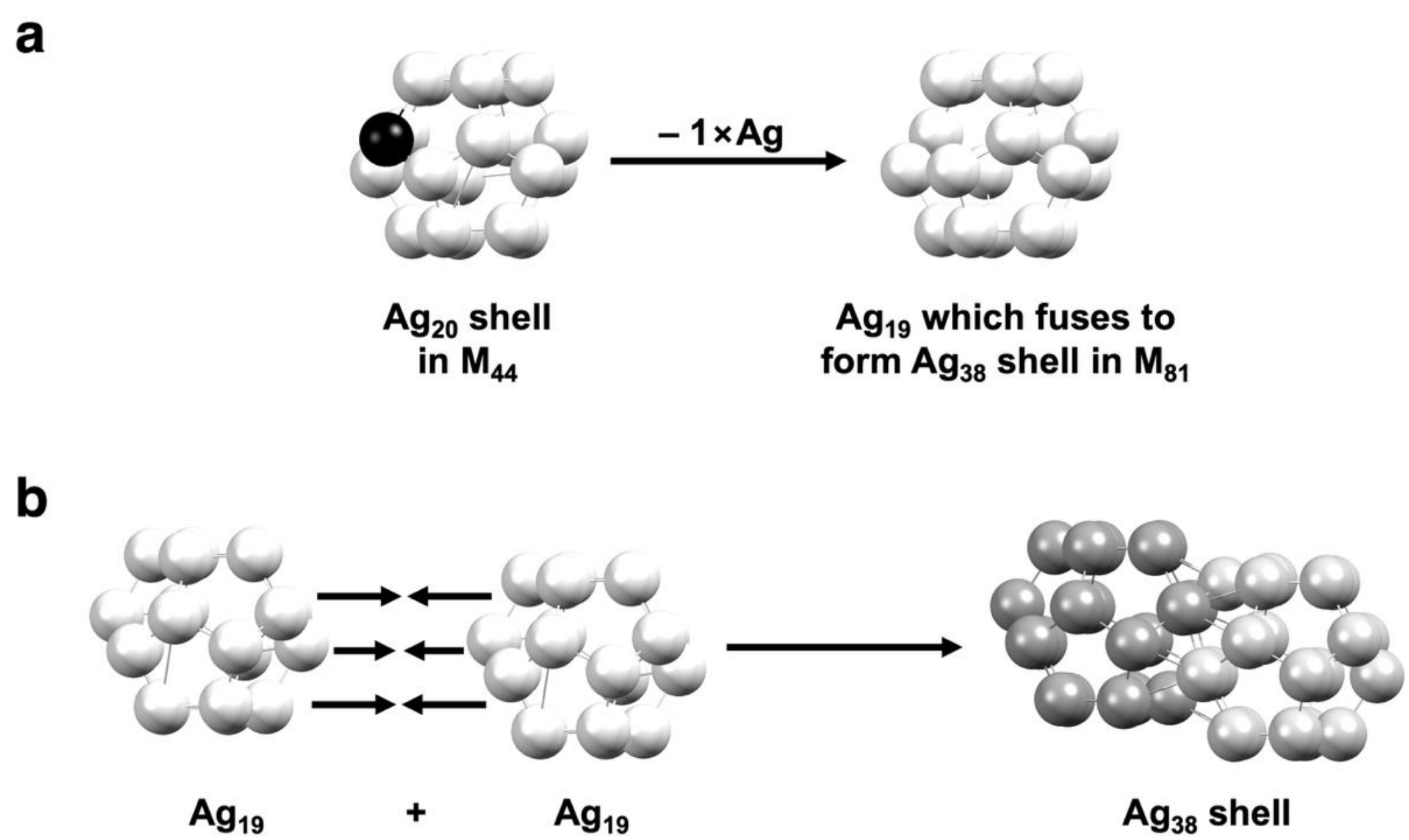

Figure S3. Formation of the Ag_{38} shell in M_{81} NCs, (a) the Ag_{20} shell in M_{44} loses one Ag atom to form Ag_{19} shell and (b) two Ag_{19} shells fuse to form the Ag_{38} shell found in M_{81}.

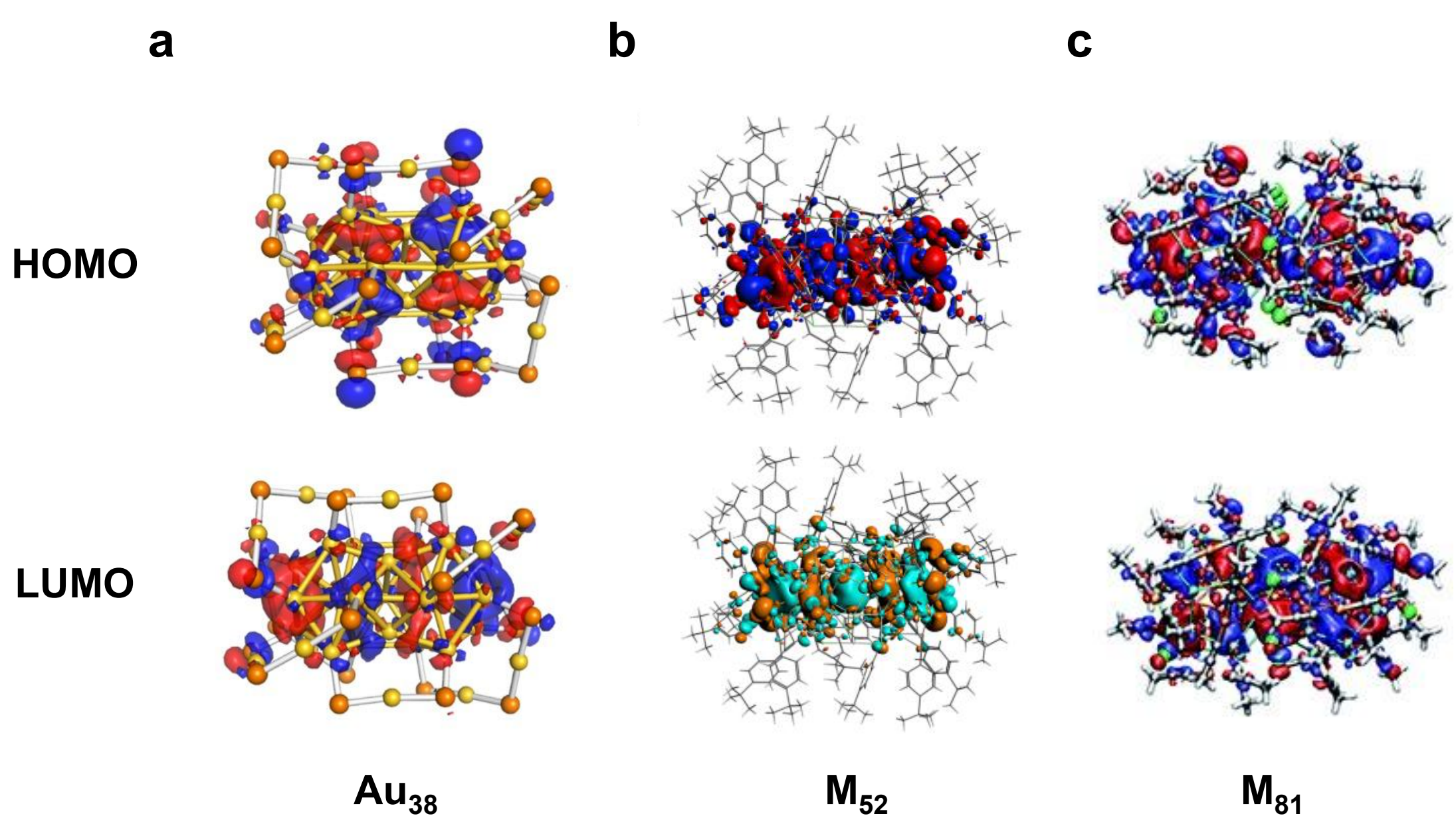

Figure S4. Distributions of the frontier molecular orbitals of (a) Au_{38}, (b) M_{52}, and (c) M_{81}. Reproduced from Refs [5], [6] and [7] with permission.

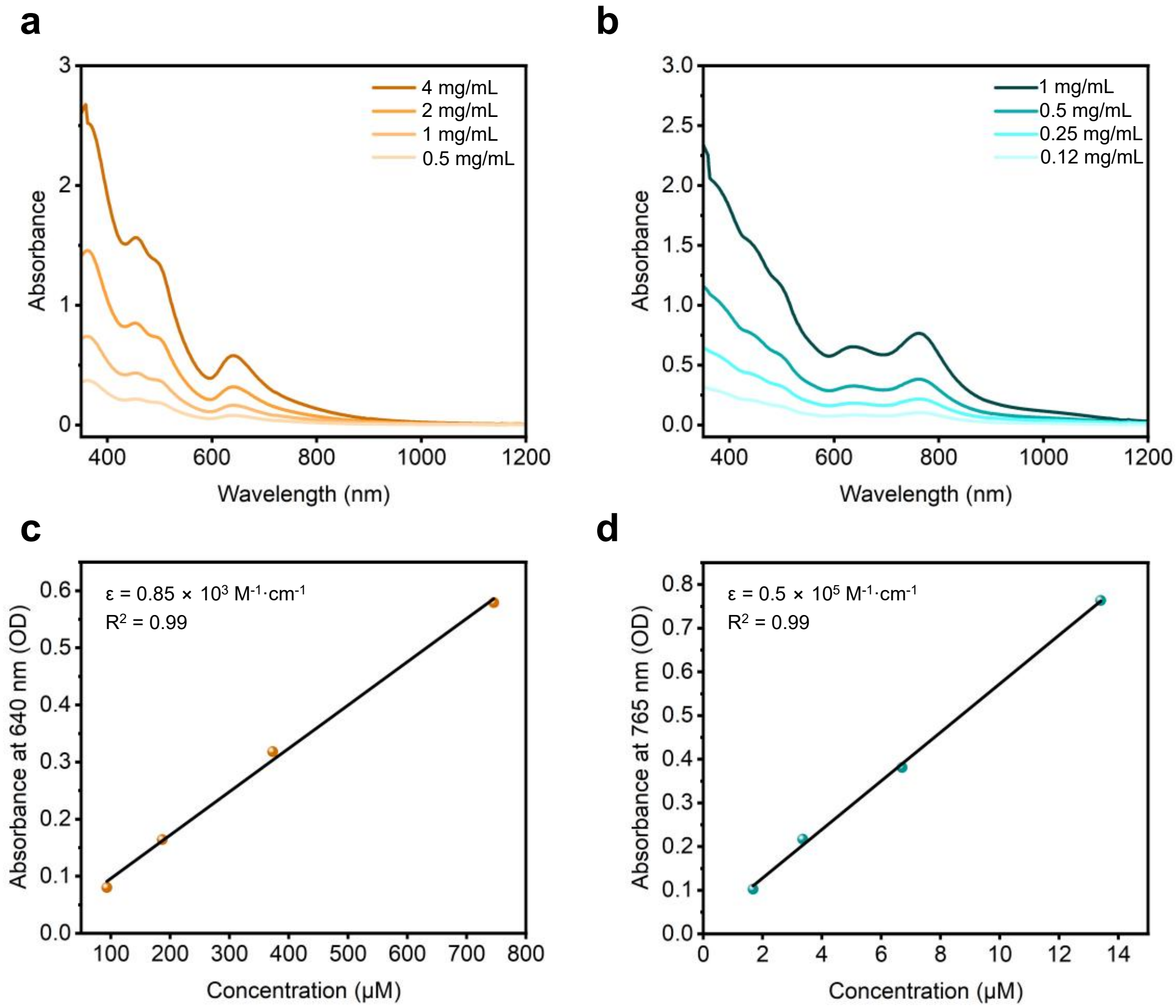

Figure S5. Absorption spectra of (a) M_{44} and (b) M_{81} NCs with different concentrations in toluene. Absorbance of (c) M_{44} and (d) M_{81} NCs vs. concentration and the linear fit at 640 nm and 765 nm, respectively, to find out the molar absorptivity or absorption coefficient (ε).

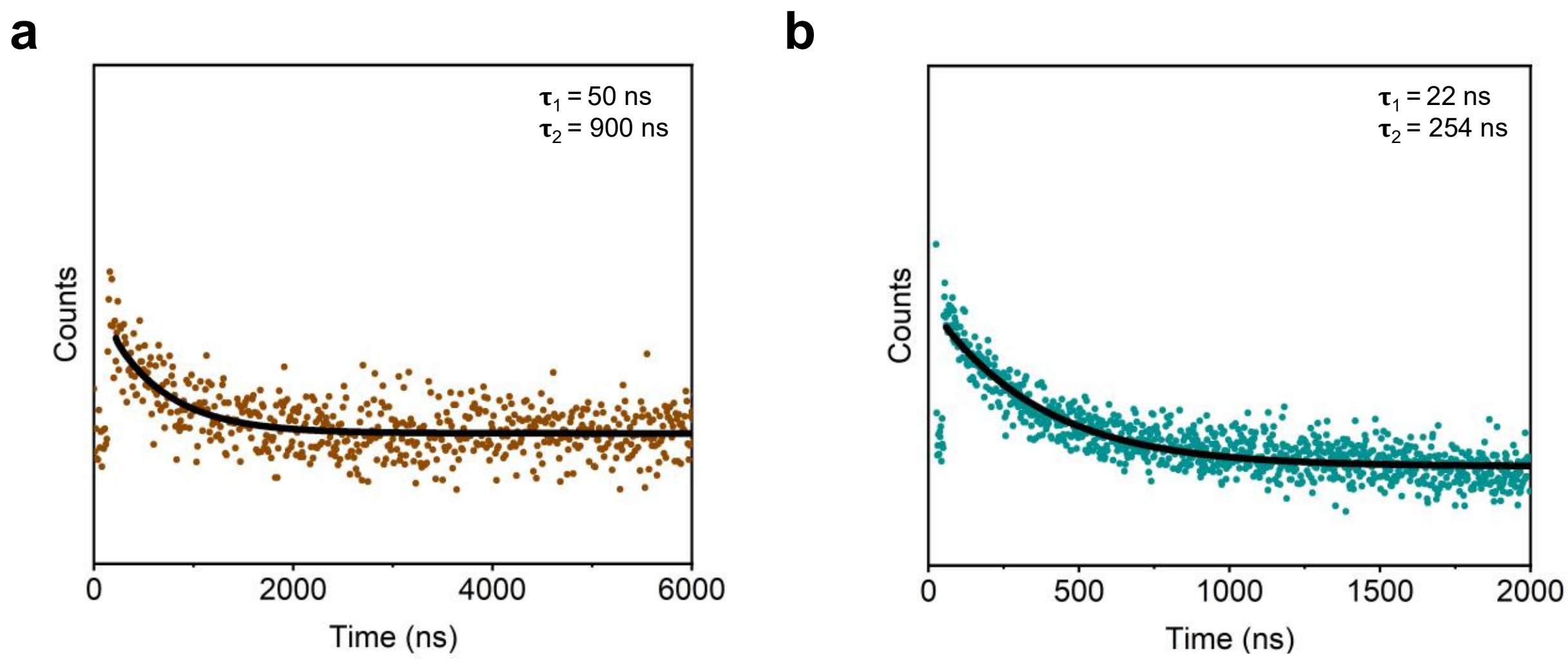

Figure S6. Emission lifetime decay profiles of (a) M_{44} and (b) M_{81} NCs in toluene.

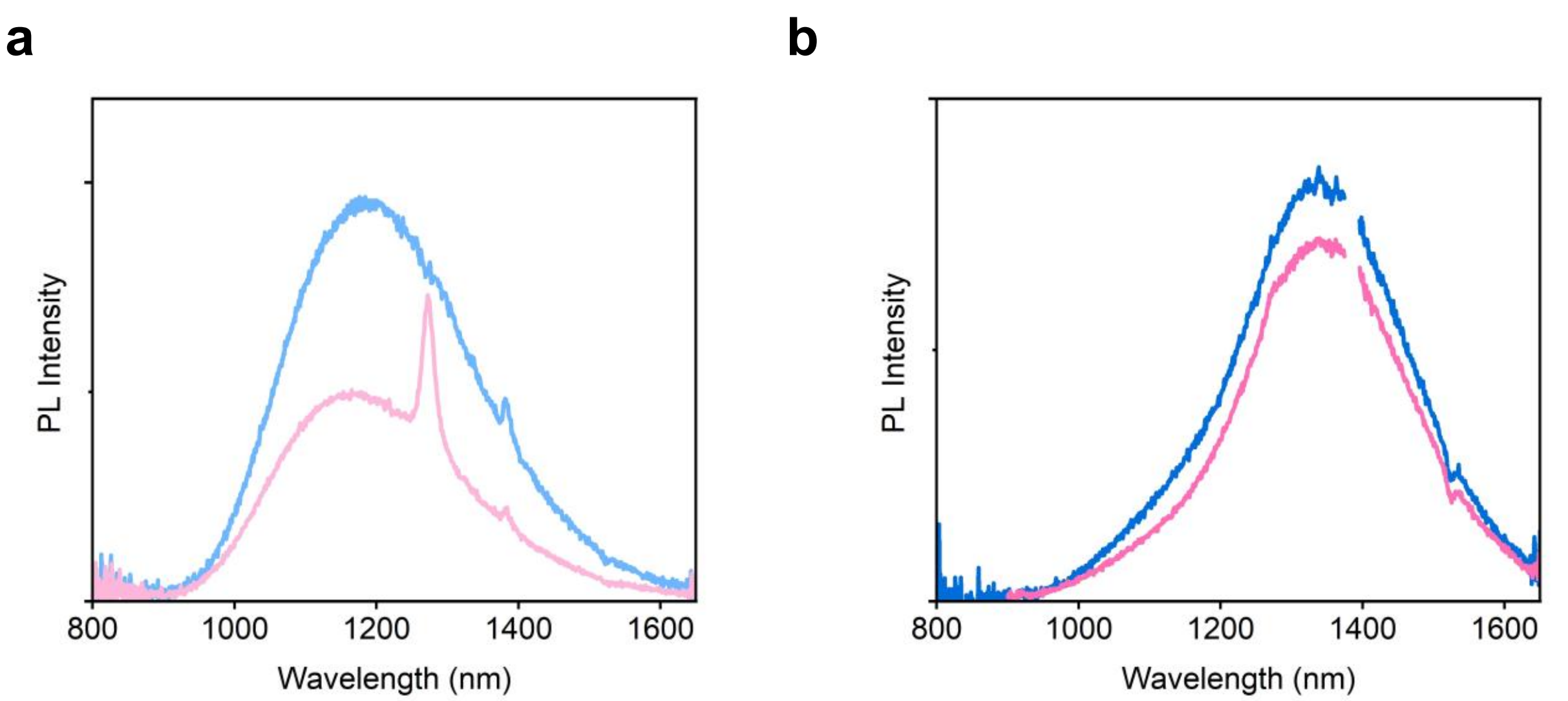

Figure S7. PL spectra under He atmosphere (blue) and O_2 atmosphere (pink) for (a) M_{44} and (b) M_{81} NCs in $CDCl_3$. *Note: for spectra in (b), the water vapor(in air)-induced instrumental artifacts at 1381 nm were removed.

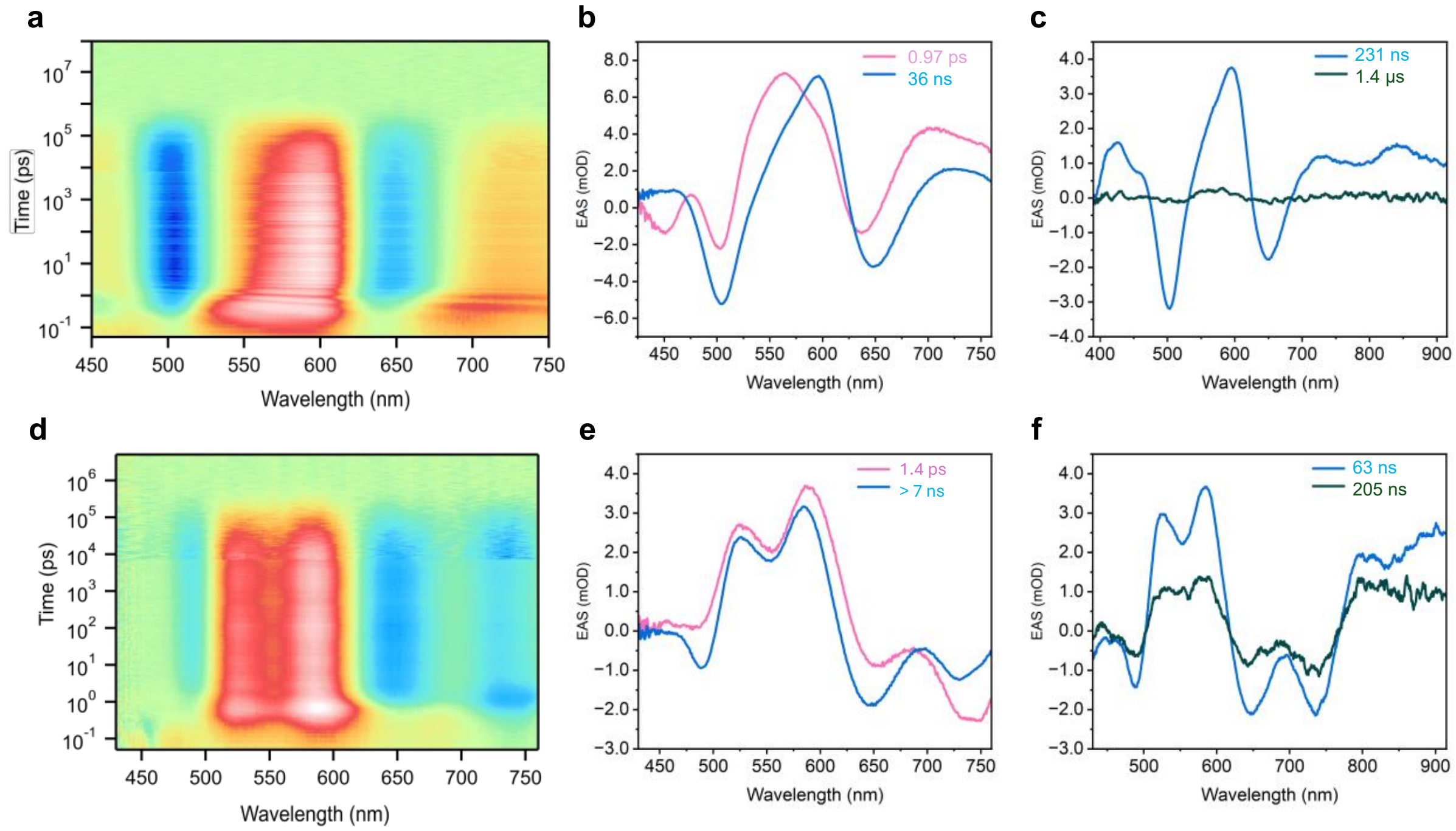

Figure S8. Transient absorption data map of (a) M_{44} and (d) M_{81} NCs in toluene pumped at 365 nm. (b, c, e and f) Global fitting analysis and evolution associated spectra for the fs-TA and ns-TA data, respectively, of M_{44} (top) and M_{81} (bottom).

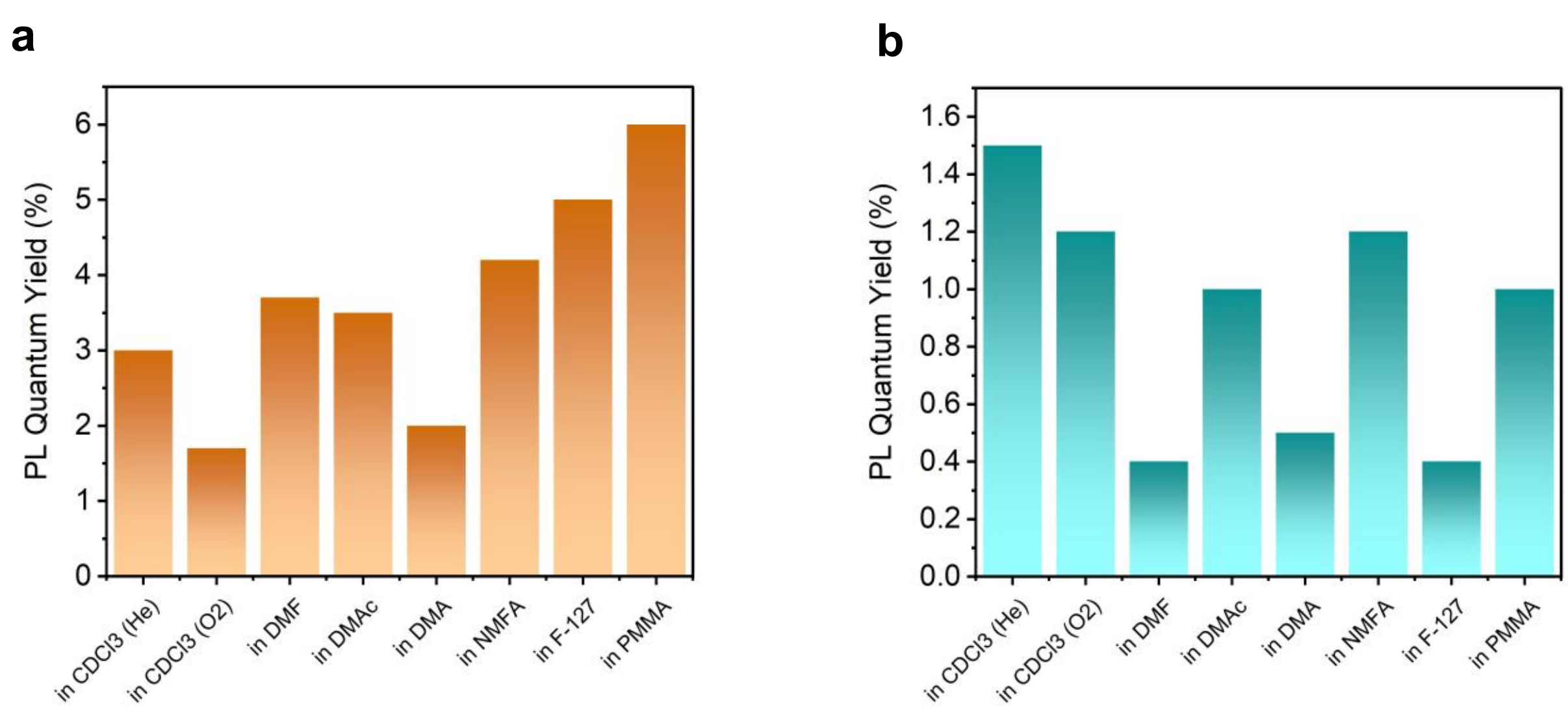

Figure S9. PL Quantum Yield (%) of (a) M_{44} and (b) M_{81} NCs measured in various solvents.

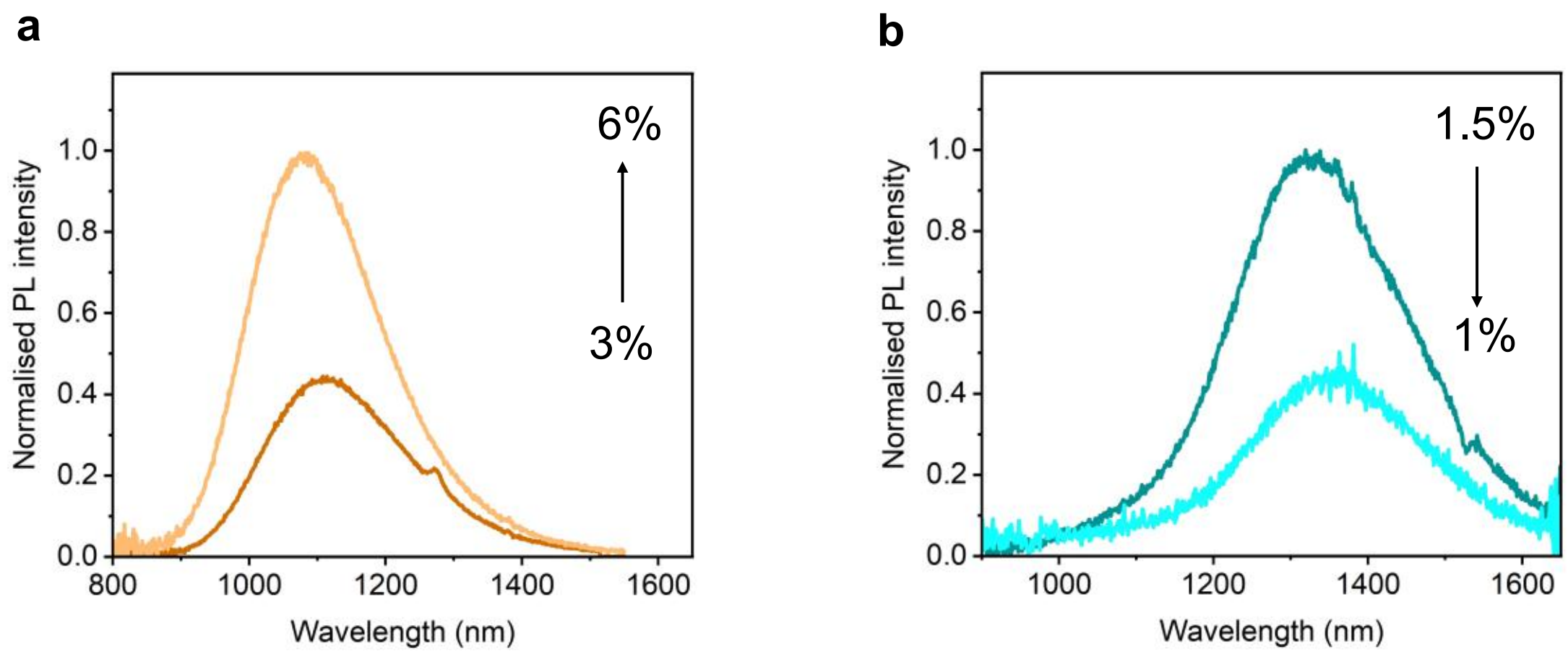

Figure S10. PL spectra of (a) M_{44} and (b) M_{81} embedded in PMMA films.

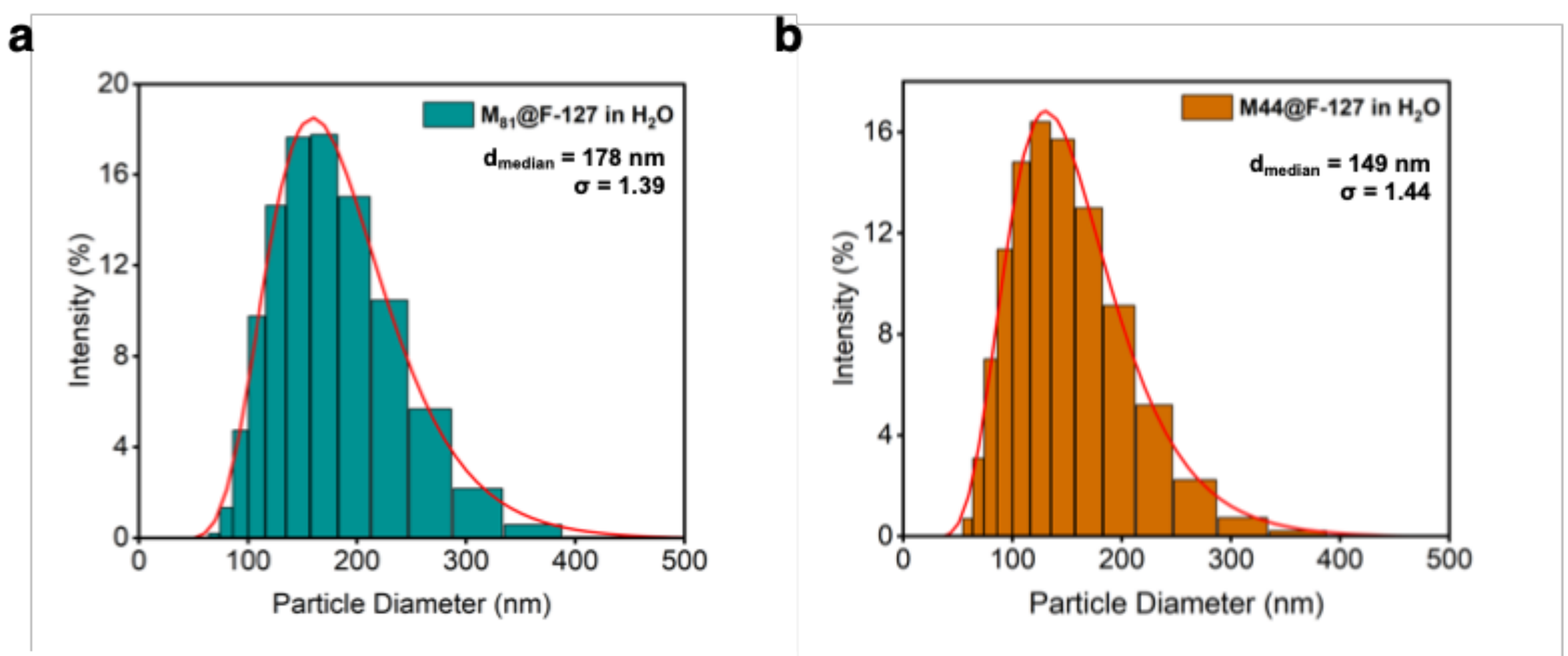

Figure S11. Dynamic Light Scattering (DLS) spectra of (a) M_{44}@F-127 and (b) M_{81}@F-127 supraparticles.

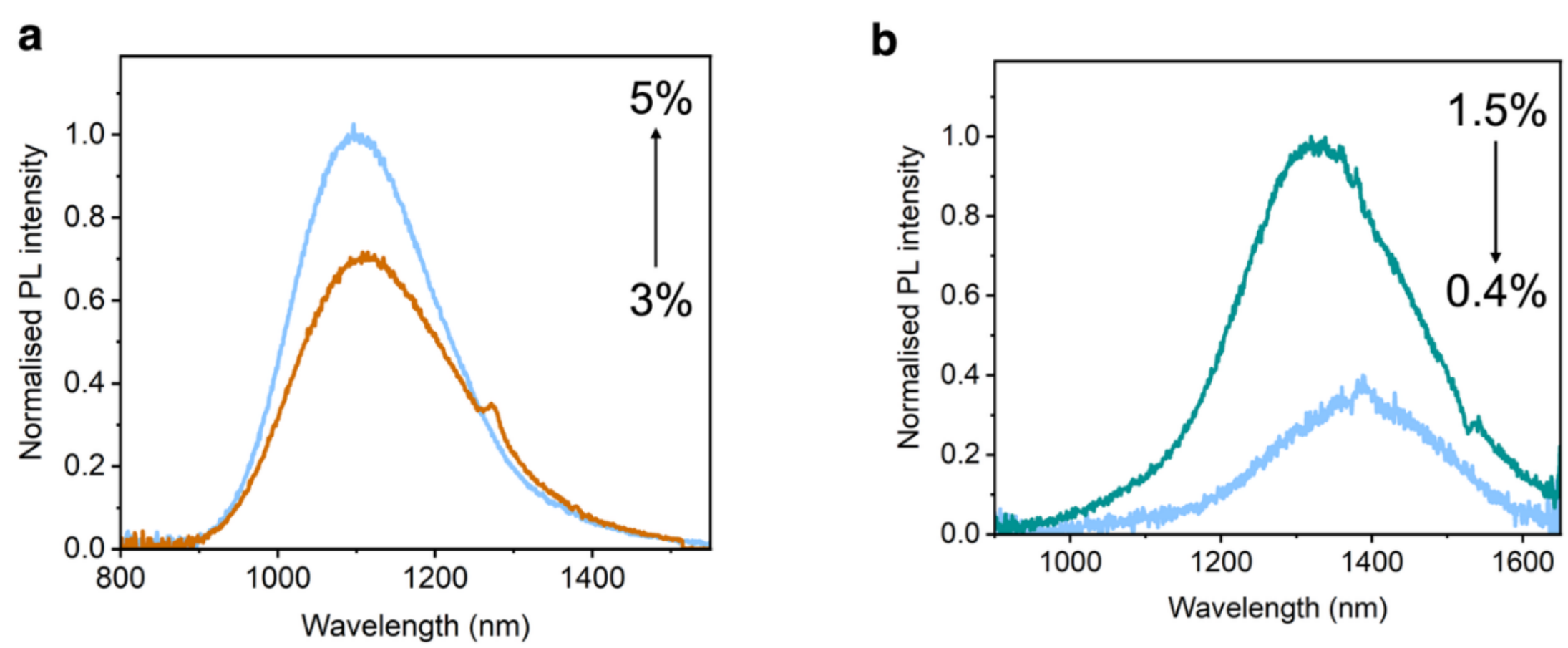

Figure S12. PL spectra of the (a) M_{44} (golden) compared to M_{44}@F-127 supraparticles (light blue) and (b) M_{81} (dark teal) compared to M_{81}@F-127 supraparticles (light blue).

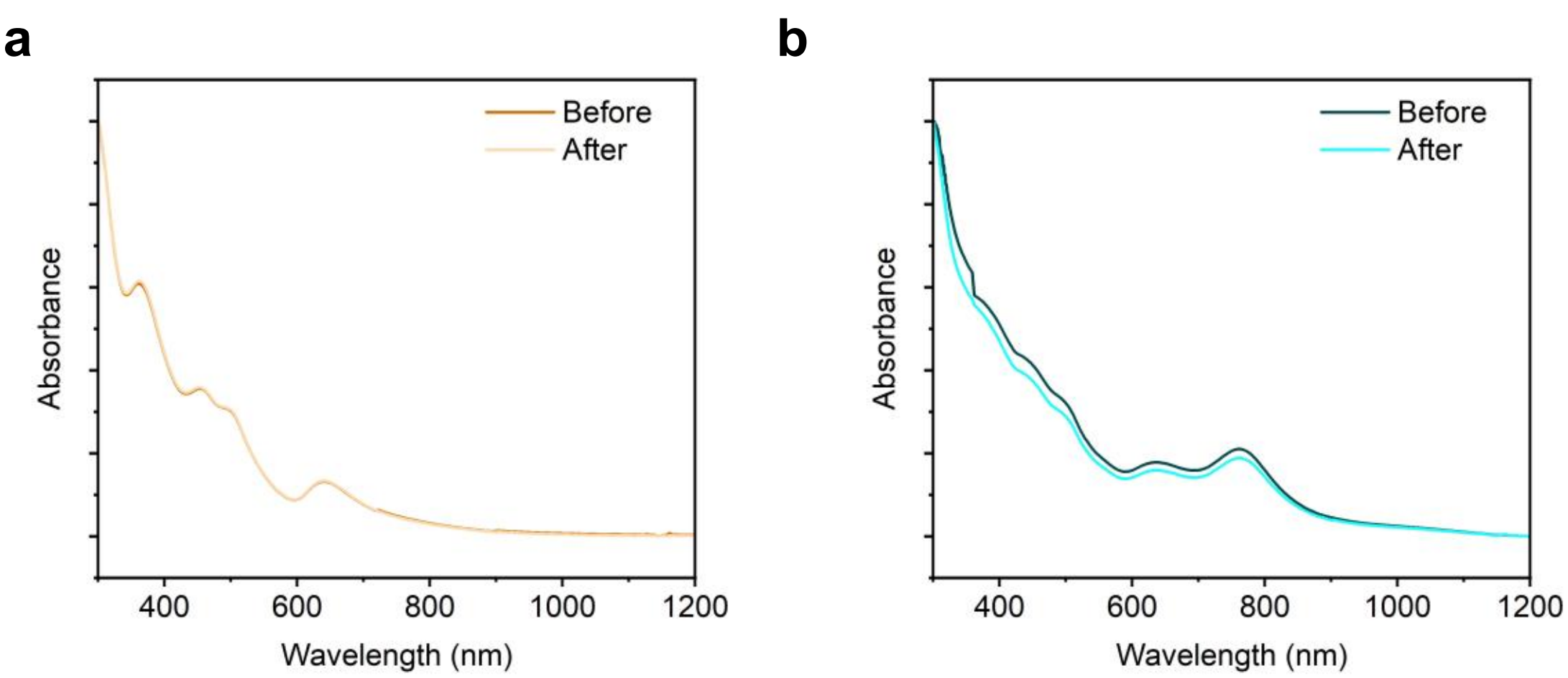

Figure S13. Stability of (a) M_{44} and (b) M_{81} NCs evaluated by comparing their UV-vis-NIR spectra before and after storage under ambient conditions for 6 months.

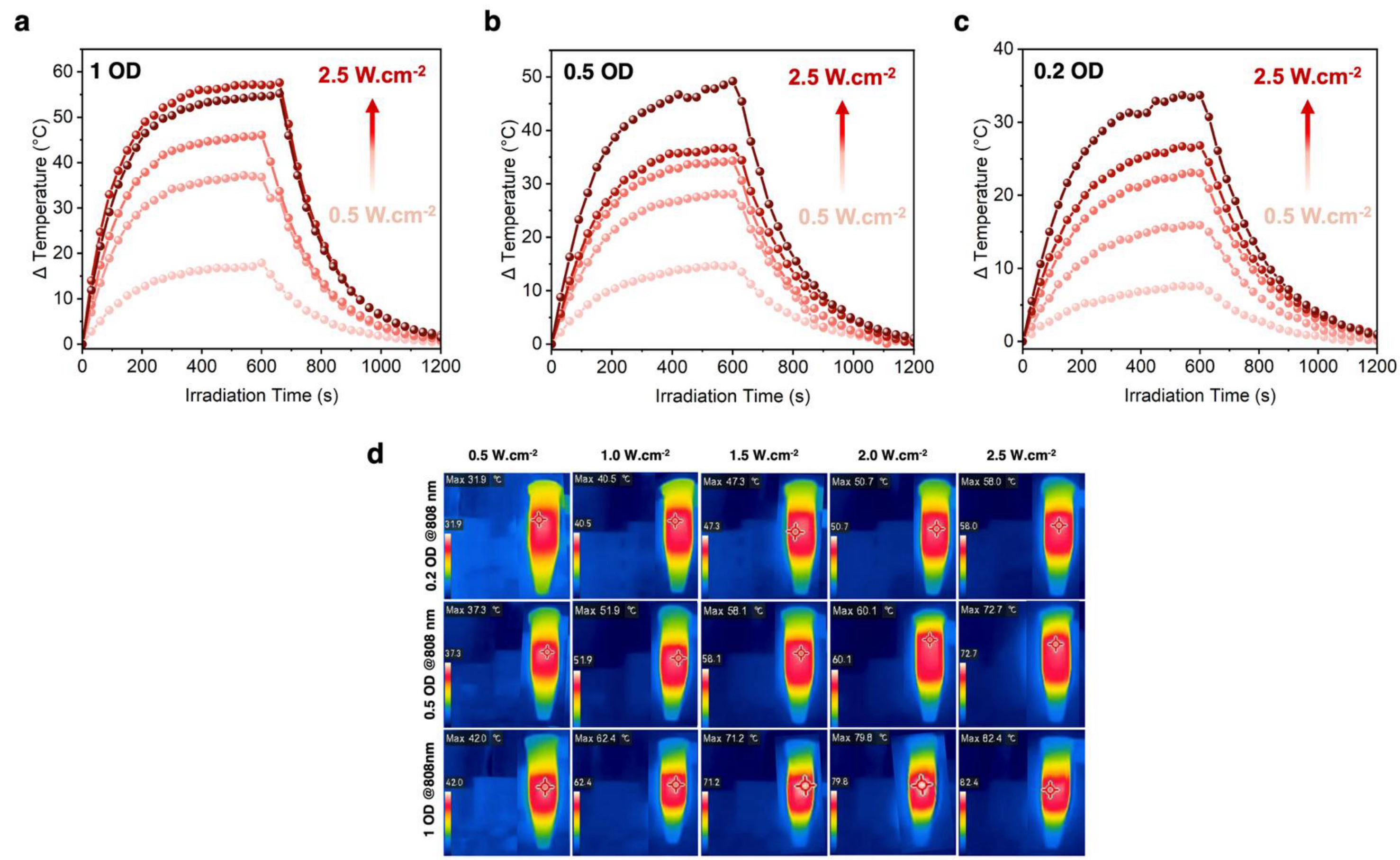

Figure S14. Heating curves/Temperature rise graph of M_{44} NCs under various power densities with absorbance around (a) 1 OD, (b) 0.5 OD and (c) 0.2 OD at 808 nm. (d) Infrared thermal camera images of the highest solution temperature at various OD and OPDs.

For example: Let's take M_{44} NCs with Abs_{808} = 0.5 OD (**A_λ**), excited by an 808 nm laser with optical power density (**I**) 0.5 W.cm^{-2} (**Figure S14c**).
From the figure, we see **ΔT_{max}** = 14.7 °C. From **Figure S15** below (top left), the linear fit of -lnθ vs t gives the slope as 138.58 s^{-1}, which corresponds to **τ_s**.
The photothermal conversion efficiency equation is, $\eta = \frac{hS\Delta T_{max}}{I\,(1-10^{-A_\lambda})}$.
We already know, $hs = \frac{\sum m_i C_{p,i}}{\tau_s}$.
For our case,
$\boldsymbol{\sum m_i C_{p,i}}$ = ρ(Toluene).V(Toluene).$C_{p(Toluene)}$ = 0.867 g mL^{-1} × 1.5 mL × 1.71 J g^{-1} ℃$^{-1}$ = 2.224 J ℃$^{-1}$.
Thus, $\boldsymbol{hs}$ = 2.224/138.58 = 0.016 J ℃$^{-1}$ s^{-1}

Plugging the values into equation $\eta = \frac{hS\Delta T_{max}}{I\,(1-10^{-A_\lambda})}$, we get

$$\eta = \frac{0.016 \times 14.7}{0.5 \times (1-10^{-0.5})} = 69\%$$

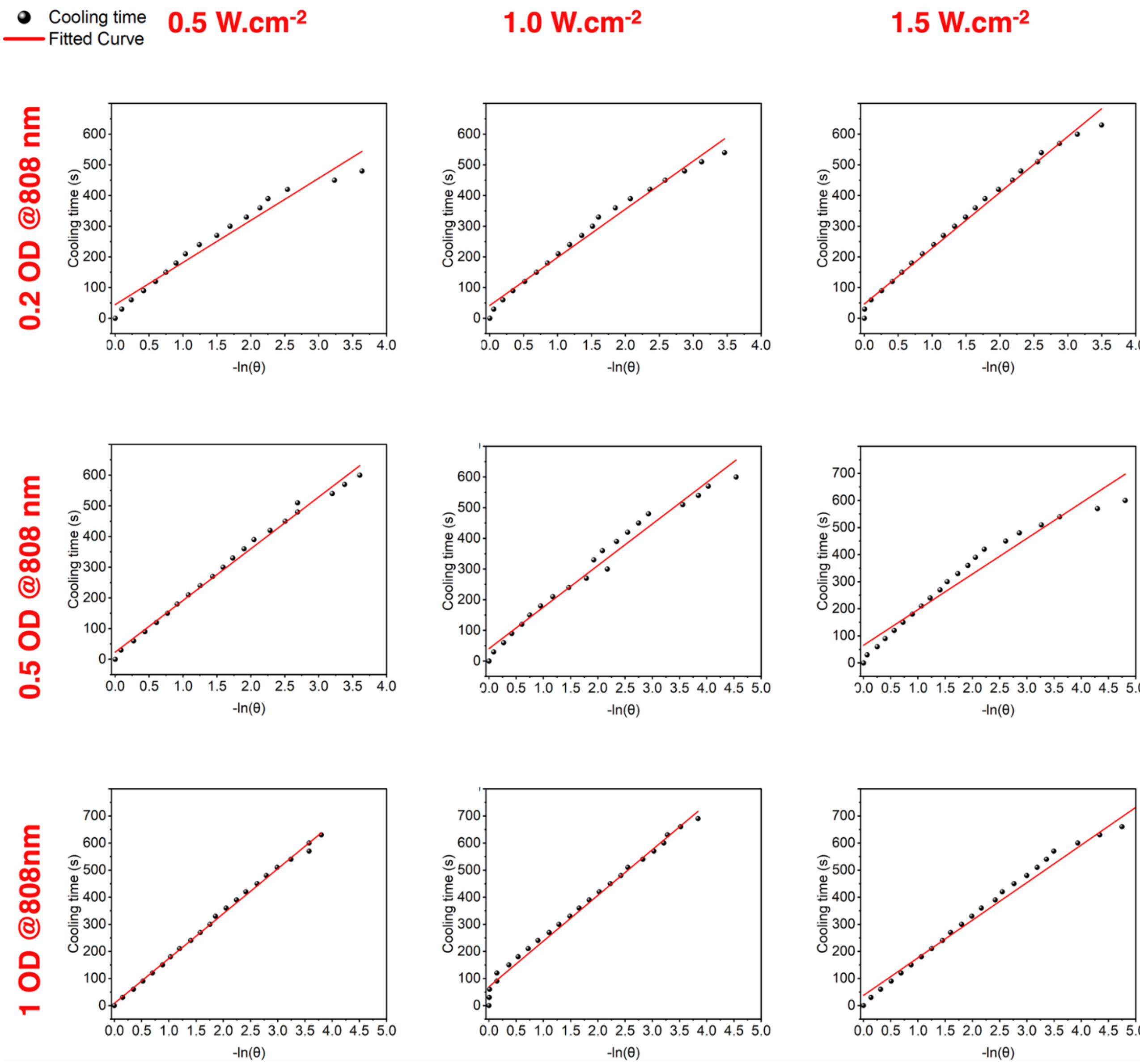

Figure S15. Plot of cooling time vs. the negative logarithm of the temperature driving force (θ) during the cooling stage for M_{44} NCs at various absorbance and OPDs during photothermal conversion experiments. (more panels continued next page)

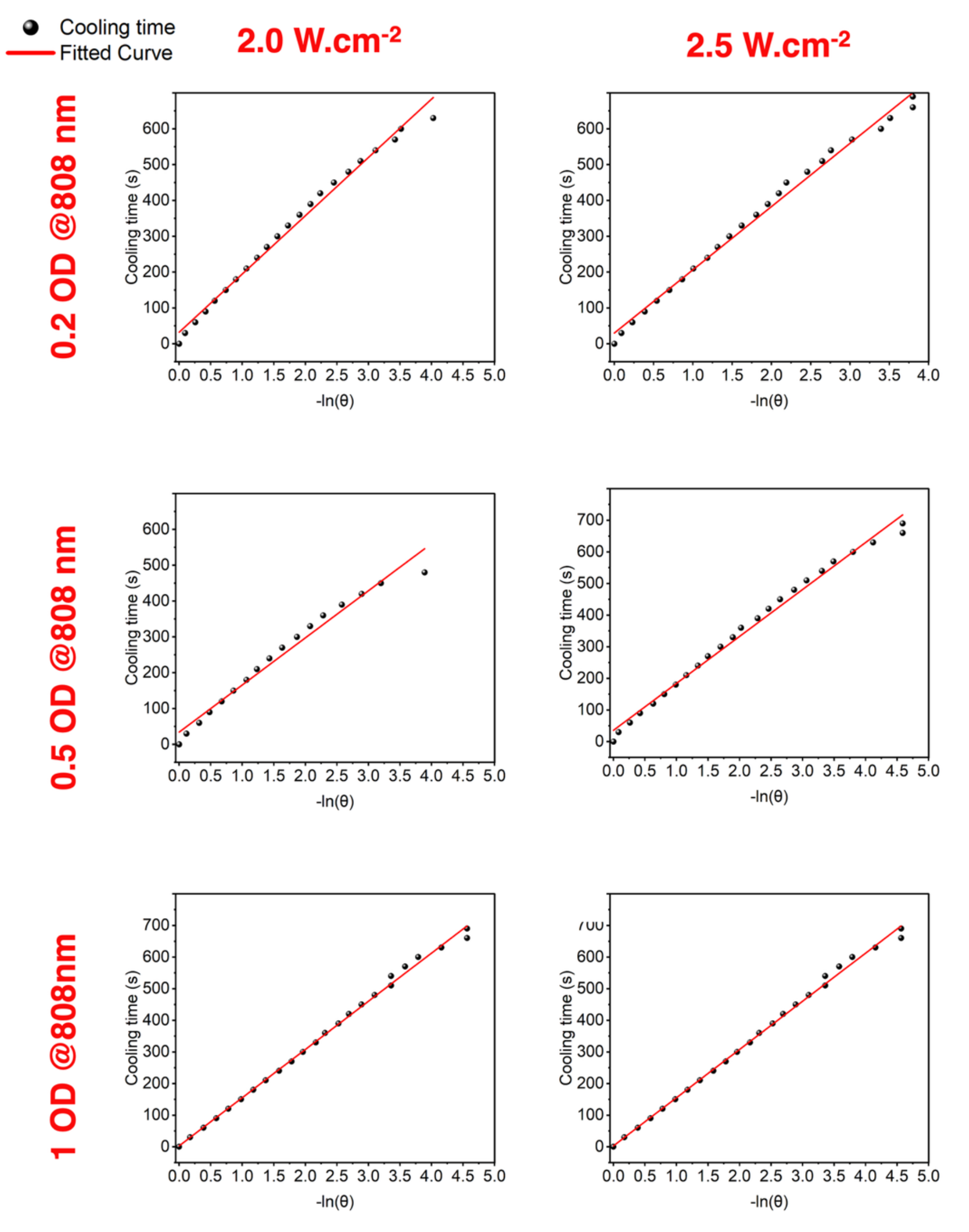

Figure S15 (continued). Plot of cooling time vs. the negative logarithm of the temperature driving force (θ) during the cooling stage for M_{44} NCs at various absorbance and OPDs during photothermal conversion experiments.

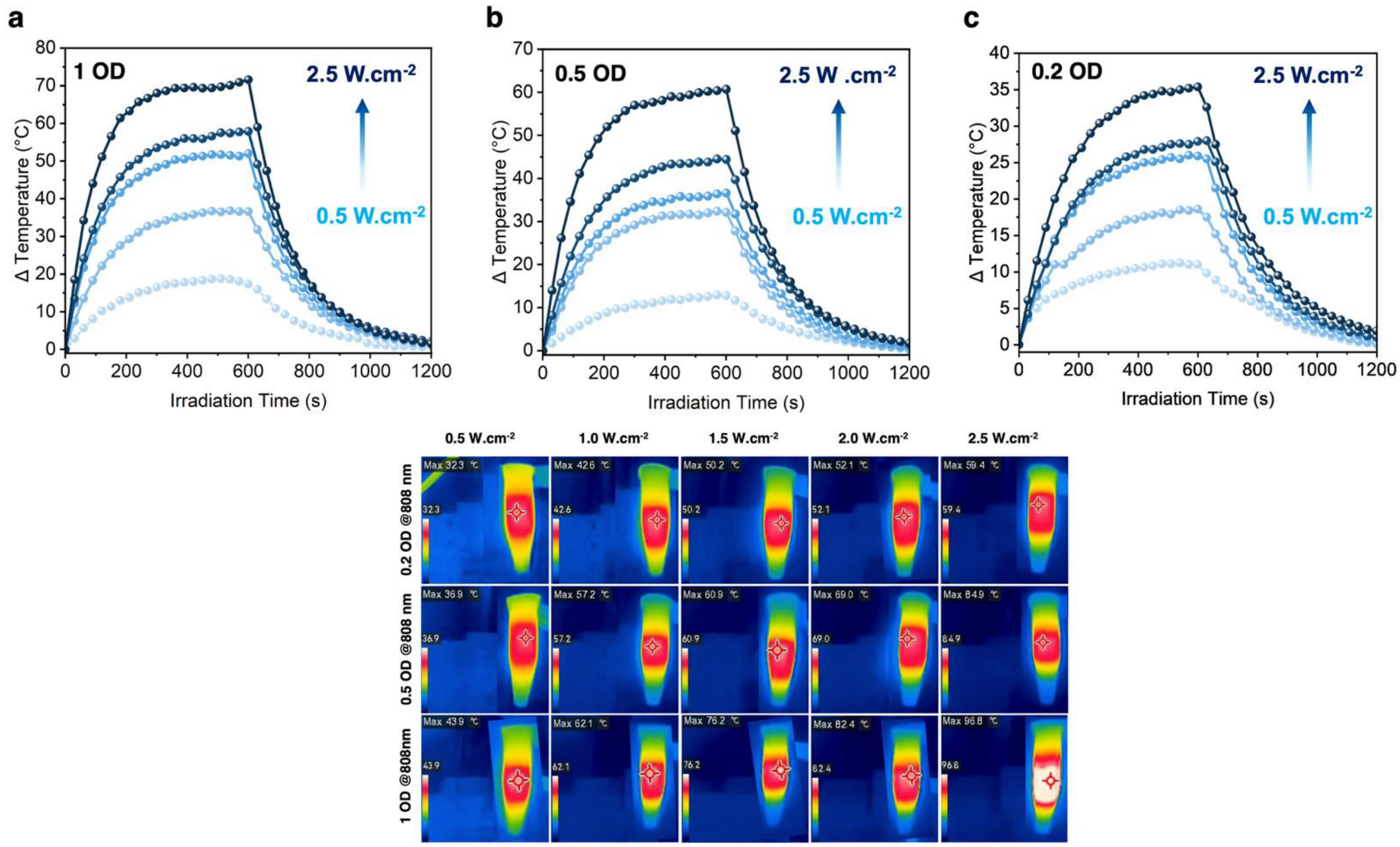

Figure S16. Heating curves/Temperature rise graph of M_{81} NCs under various power densities with absorbance (a) 1 OD, (b) 0.5 OD and (c) 0.2 OD at 808nm. Infrared thermal camera images of the highest solution temperature at various OD and OPDs.

For example: Let's take M_{81} NCs with Abs_{808} = 0.2 OD (**A_λ**), excited by an 808 nm laser with optical power density (**I**) 0.5 W.cm^{-2} (**Figure S16c**).
From the figure, we see **ΔT_{max}** = 11.2 °C. From the **Figure S17** below (top left), linear fit of -lnθ vs t gives the slope as 147.25 s^{-1}, which corresponds to **τ_s**.
The photothermal conversion efficiency equation is, $\eta = \frac{hS\Delta T_{max}}{I\,(1-10^{-A_\lambda})}$.
We already know, $hs = \frac{\sum m_i C_{p,i}}{\tau_s}$.
For our case,
$\boldsymbol{\sum m_i C_{p,i}}$ = ρ(Toluene).V(Toluene).$C_{p(Toluene)}$ = 0.867 g mL^{-1} × 1.5 mL × 1.71 J g^{-1} °C^{-1} = 2.224 J °C^{-1}.
Thus, $\boldsymbol{hs}$ = 2.224/147.35 = 0.0151 J °C^{-1} s^{-1}

Plugging the values into equation $\eta = \frac{hS\Delta T_{max}}{I\,(1-10^{-A_\lambda})}$, we get

$$\eta = \frac{0.0151 \times 11.2}{0.5 \times (1-10^{-0.2})} = 91\%$$

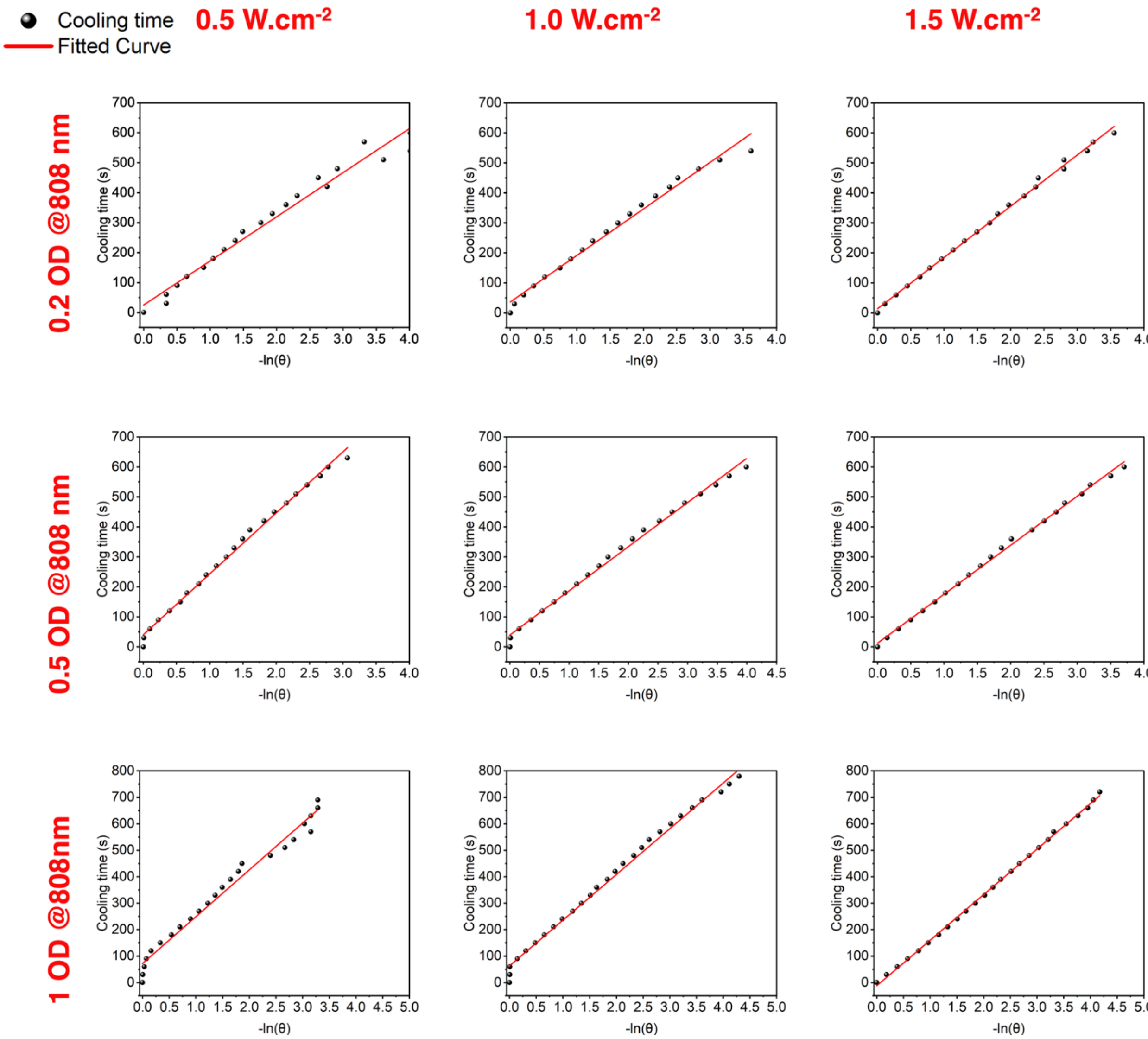

Figure S17. Plot of cooling time vs. the negative logarithm of the temperature driving force (θ) during the cooling stage for M_{81} NCs at various absorbance and OPDs during photothermal conversion experiments. (more panels continued next page)

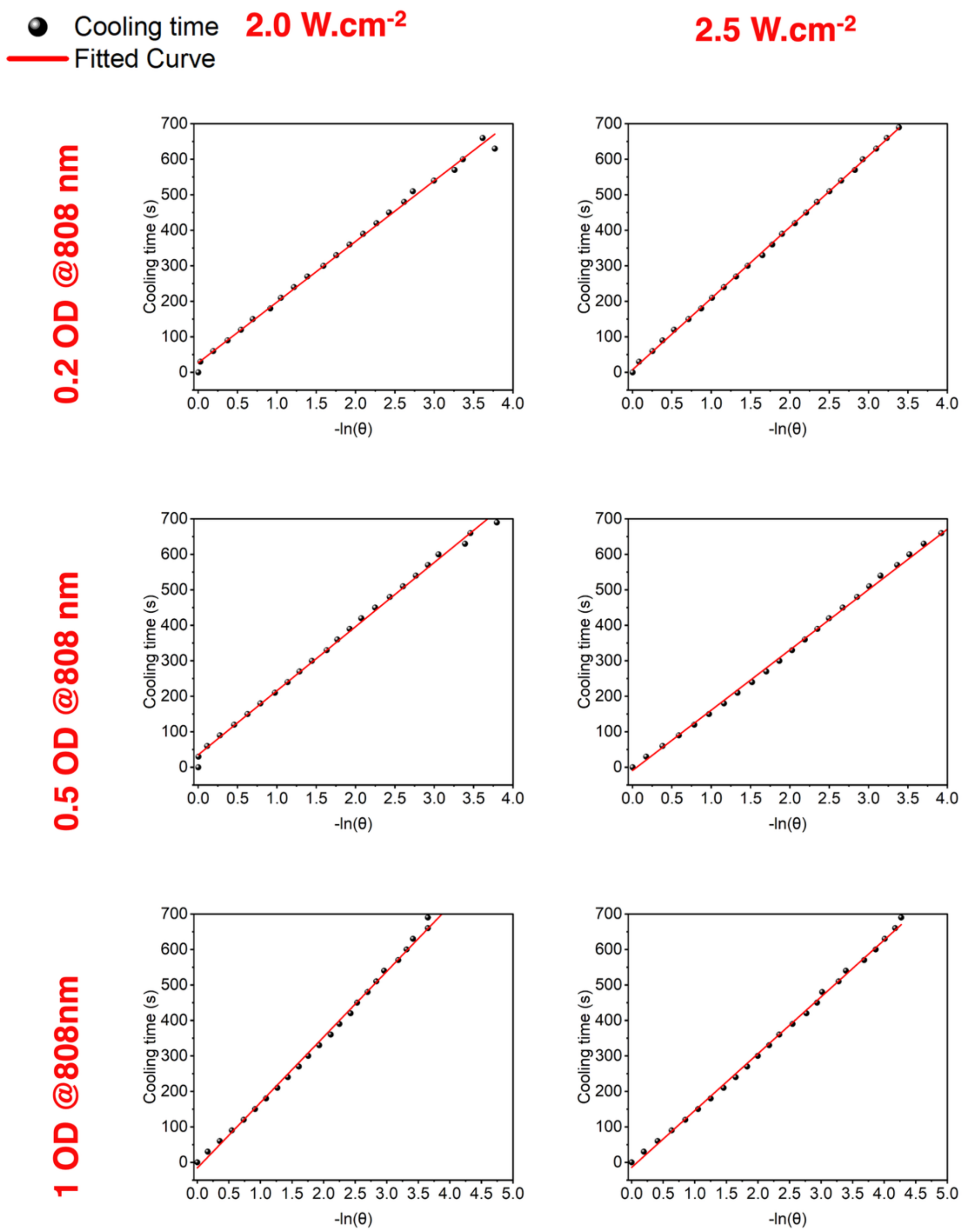

Figure S17 (continued). Plot of cooling time vs. the negative logarithm of the temperature driving force (θ) during the cooling stage for M_{81} NCs at various absorbance and OPDs during photothermal conversion experiments.

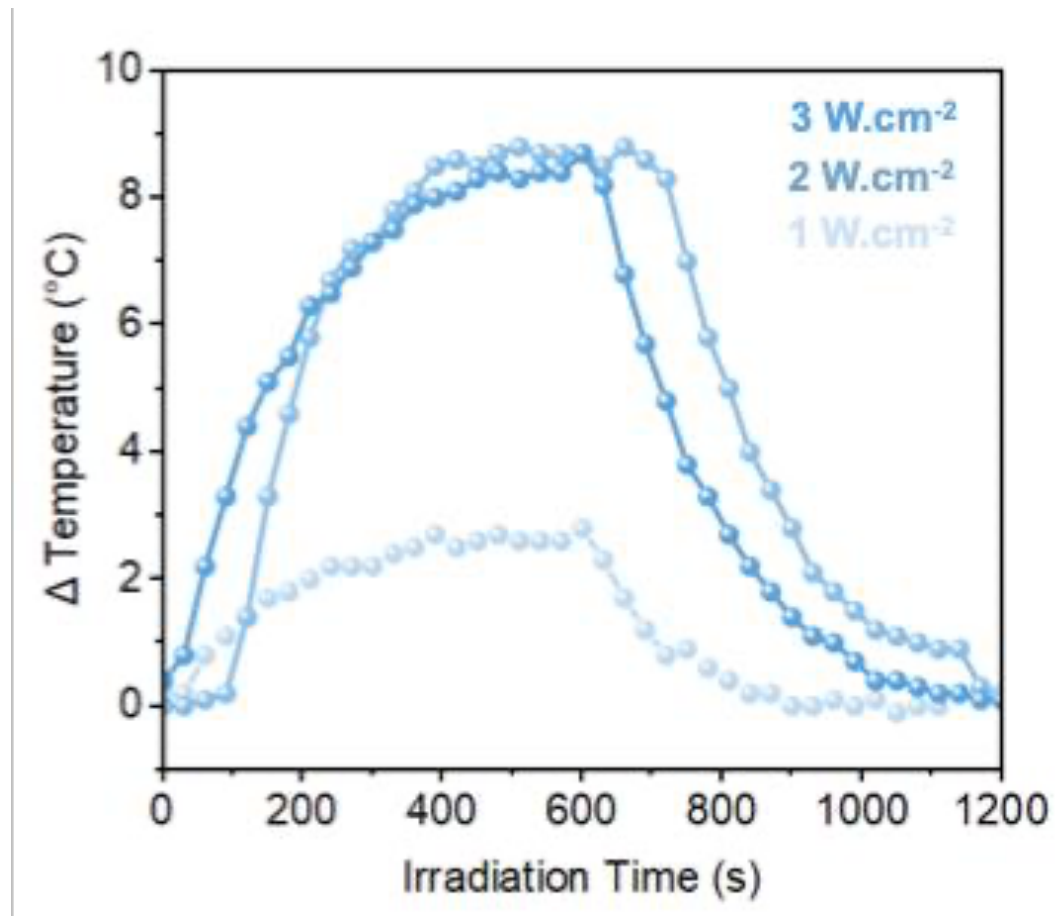

Figure S18. Heating curves/Temperature rise graph for 0.1 OD @1064nm of M_{81} NCs under various power densities irradiated with a NIR-II 1064 nm laser.

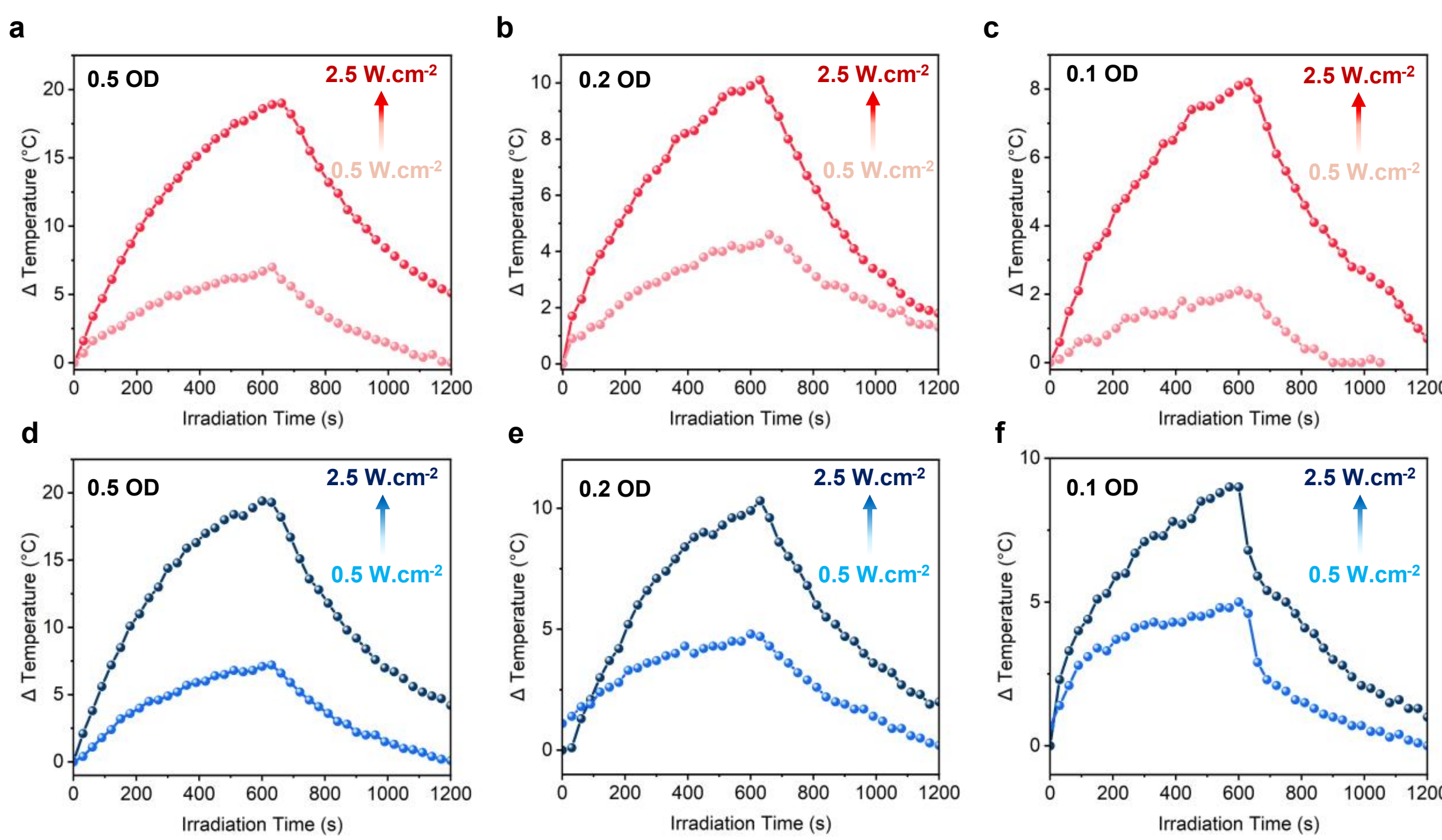

Figure S19. Heating curves/Temperature rise graph of M_{44}@F-127 supraparticles under various power densities with absorbance around (a) 0.5 OD, (b) 0.2 OD and (c) 0.1 OD at 808nm. Similar curves for the case of M_{81}@F-127 supraparticles under various power densities with absorbance around (d) 0.5 OD, (e) 0.2 OD and (f) 0.1 OD at 808nm.

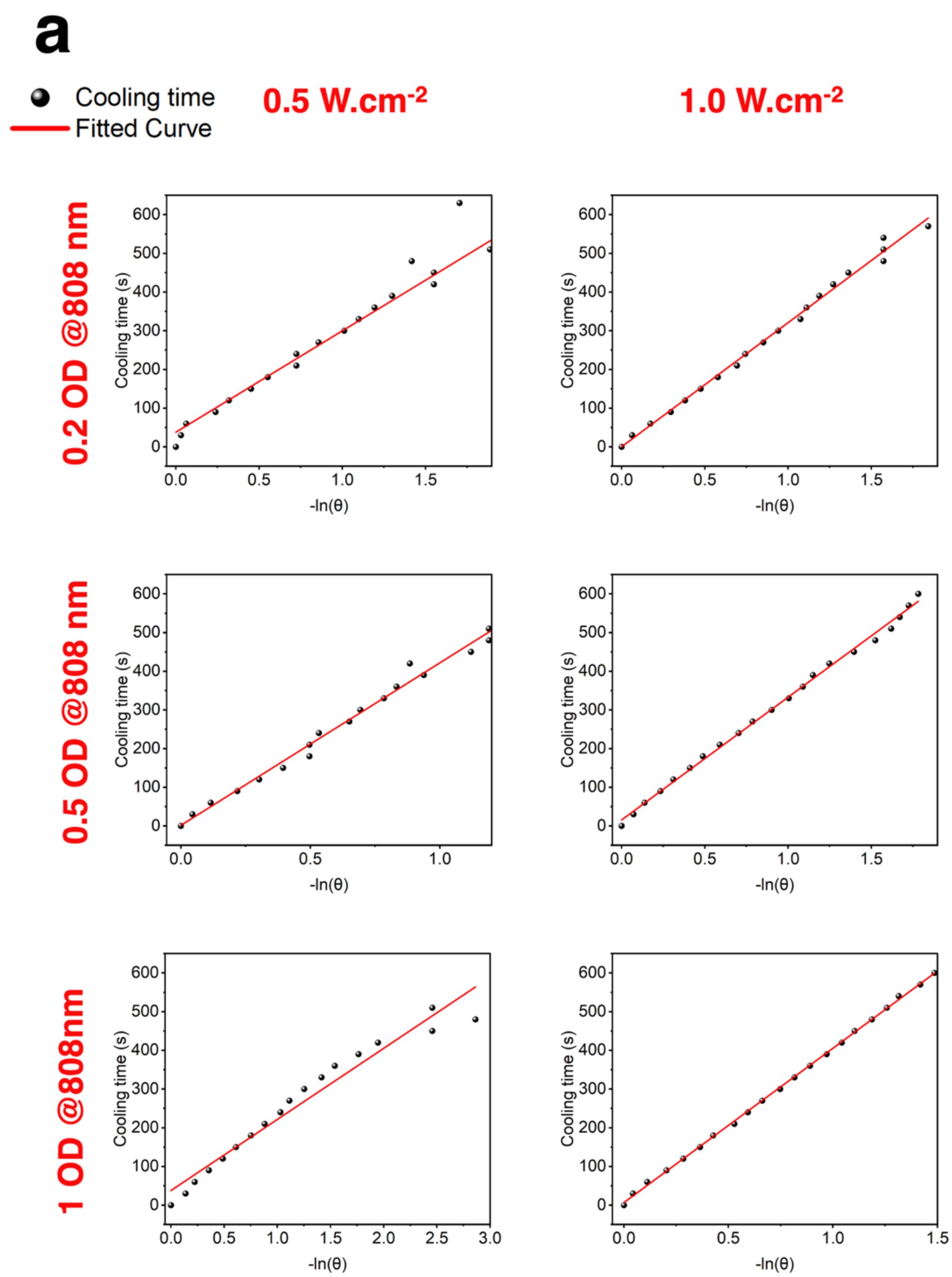

Figure S20a. Plot of cooling time vs. the negative logarithm of the temperature driving force (θ) during the cooling stage for M_{44}@F-127 supraparticles. (more panels continued next page)

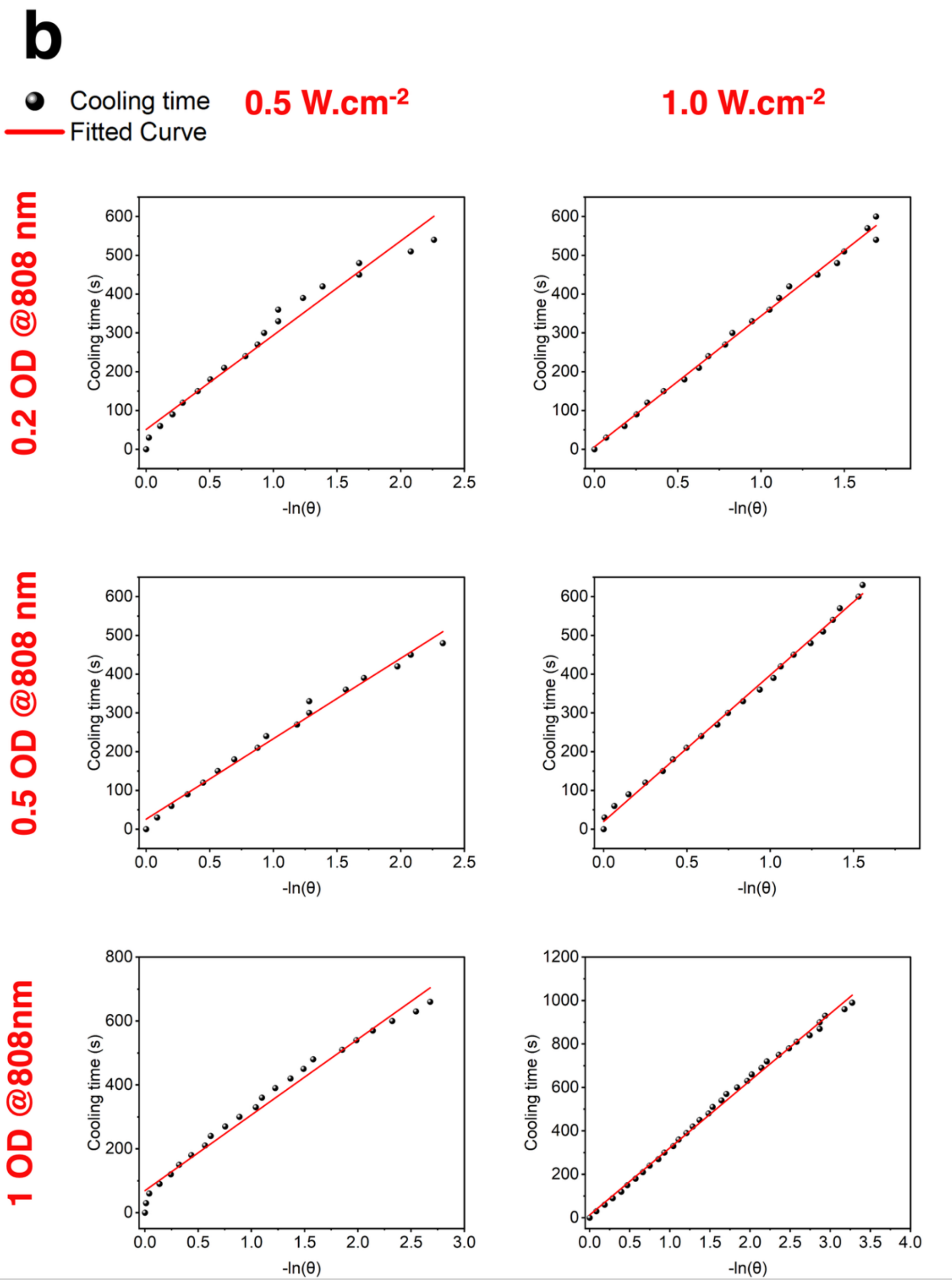

Figure S20b. Plot of cooling time vs. the negative logarithm of the temperature driving force (θ) during the cooling stage for M_{81}@F-127 supraparticles at various absorbance and OPDs during photothermal conversion experiments.

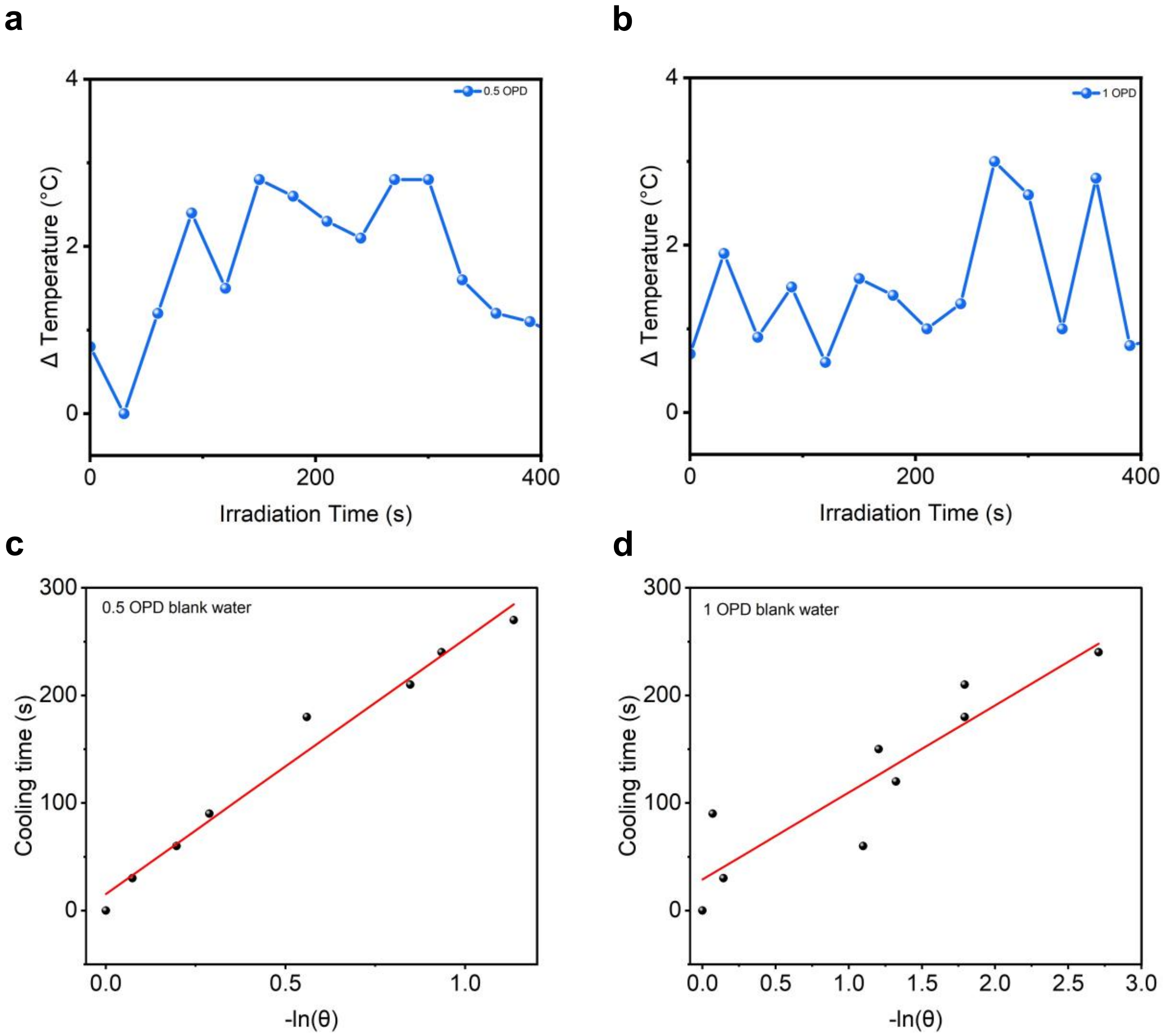

Figure S21. Heating curves of blank water irradiated with 808 nm laser at OPDs of (a) 0.5 W·cm^{-2} and (b) 1 W·cm^{-2}. Plot of cooling time vs. the negative logarithm of the temperature driving force (θ) during the cooling stage of water irradiation with 808 nm laser at OPDs of (a) 0.5 W·cm^{-2} and (b) 1 W·cm^{-2}.

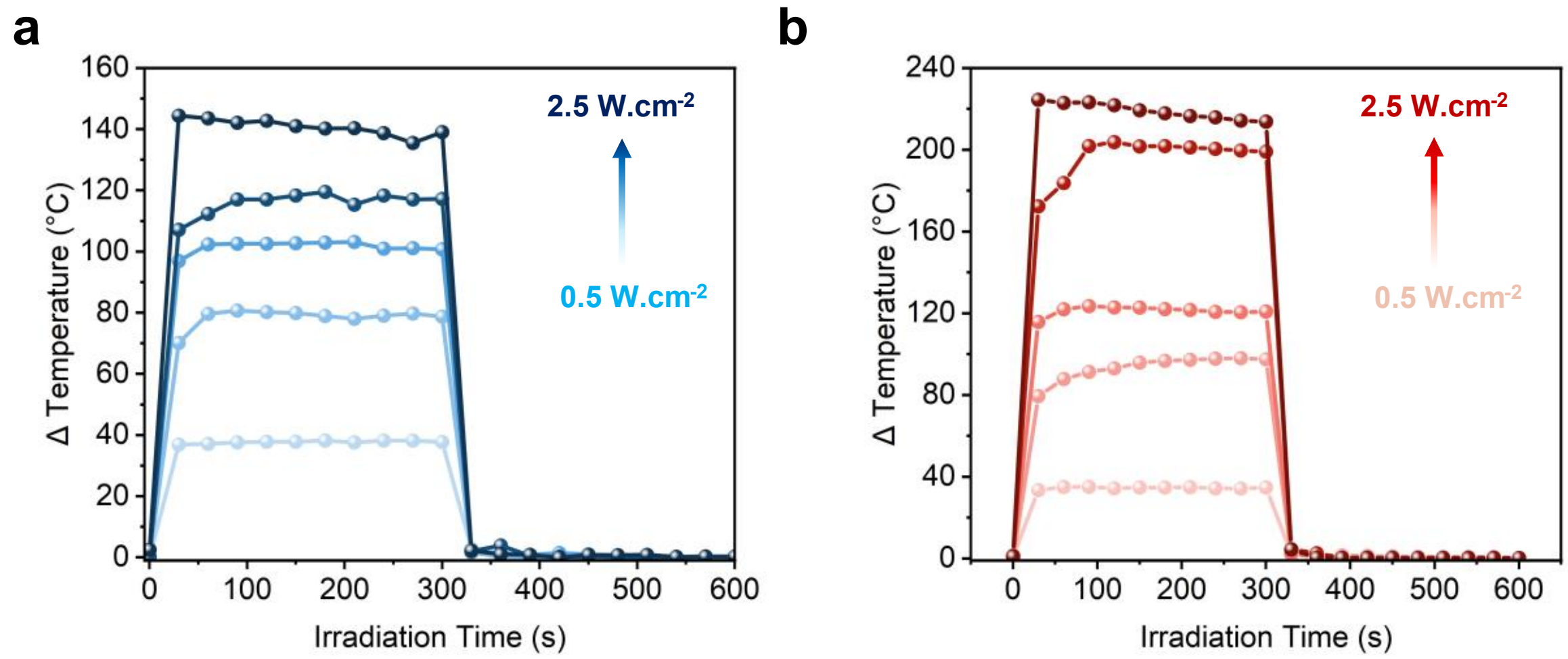

Figure S22. Heating curves/Temperature rise graph of (a) M_{81}@PMMA films and (b) M_{44}@PMMA films under various OPDs.

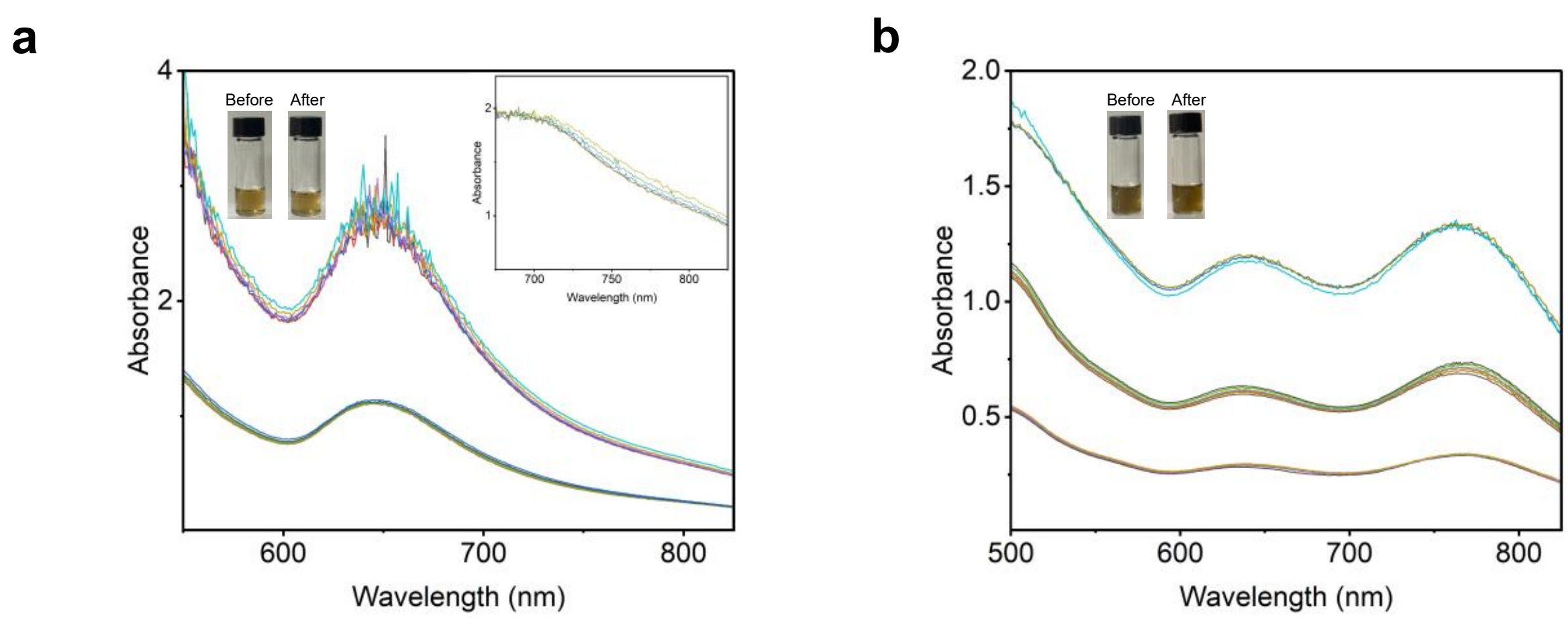

Figure S23. Photostability of (a) M_{44} and (b) M_{81} NCs in toluene before and after photothermal conversion experiments under various conditions. In (a), the higher concentration experiments are shown inset due to UV-vis-NIR detector saturation. Both the figures show images of the NCs before and after laser irradiation.

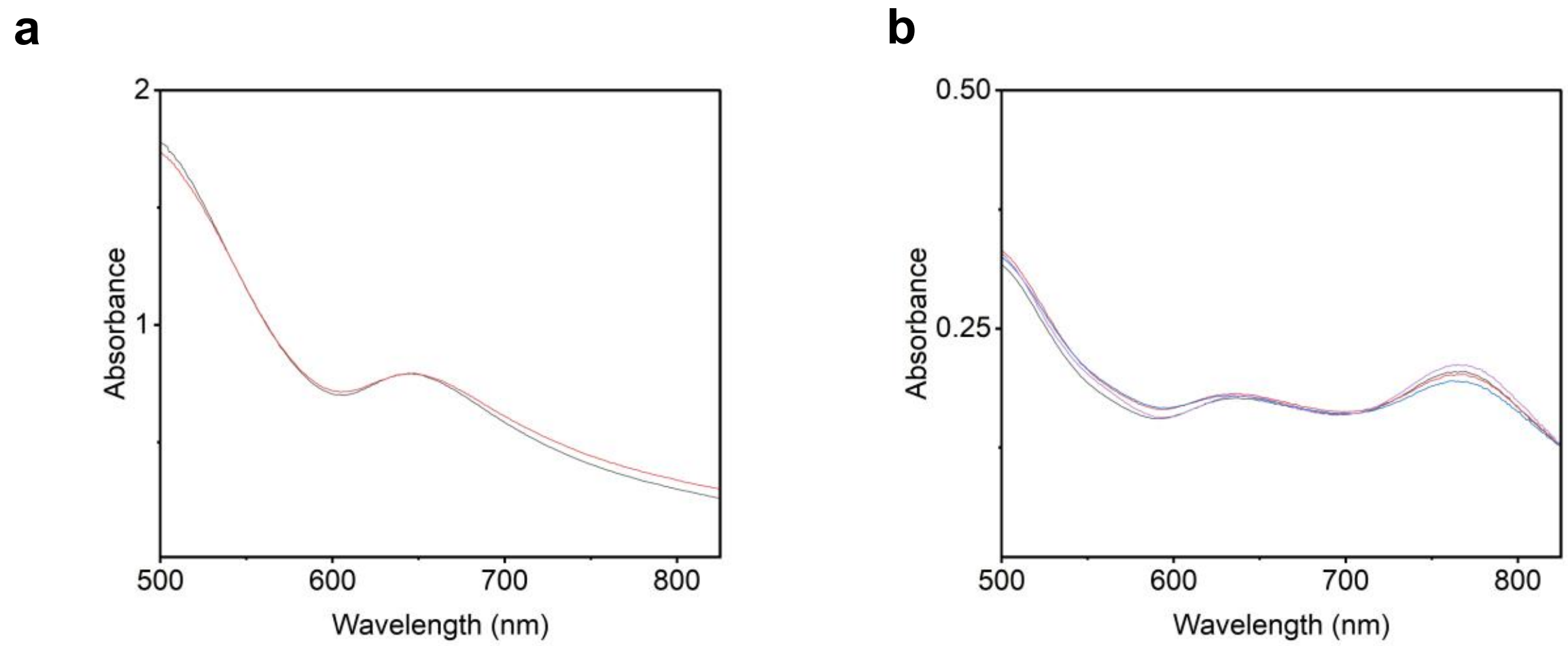

Figure S24. Photostability of (a) M_{44}@F-127 supraparticles and (b) M_{81}@F-127 supraparticles in water before and after photothermal conversion experiments under various conditions.

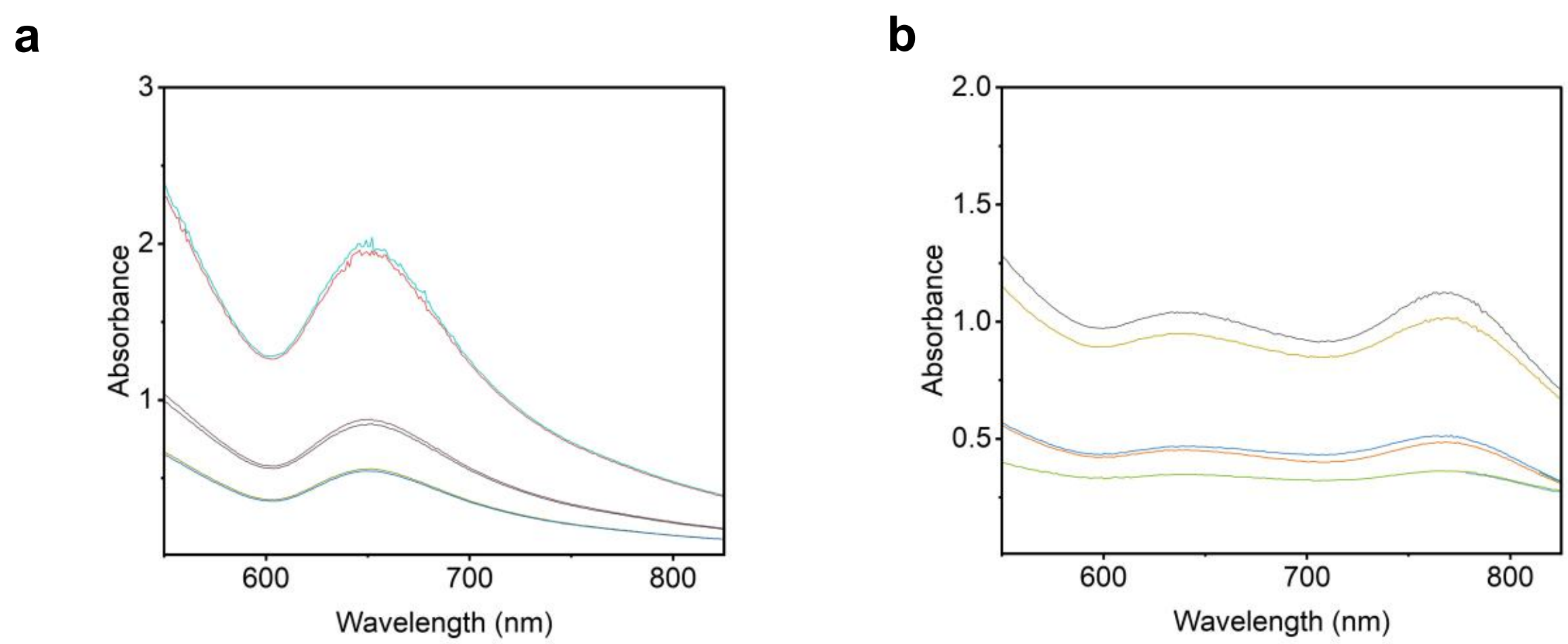

Figure S25. Photostability of (a) M_{44}@PMMA films and (b) M_{81}@PMMA films before and after photothermal conversion experiments under various conditions.

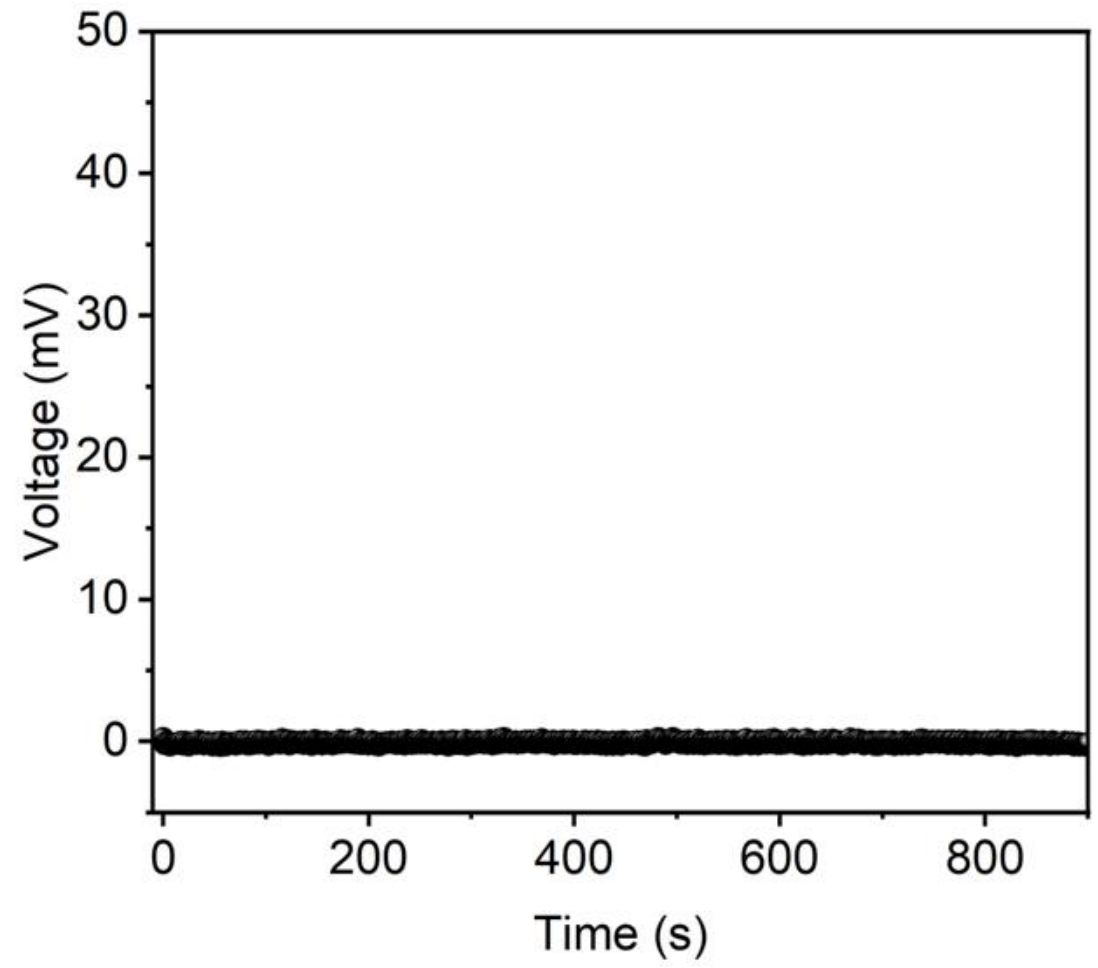

Figure S26. Open-circuit voltage generated through blank TEG device under 808 nm irradiation

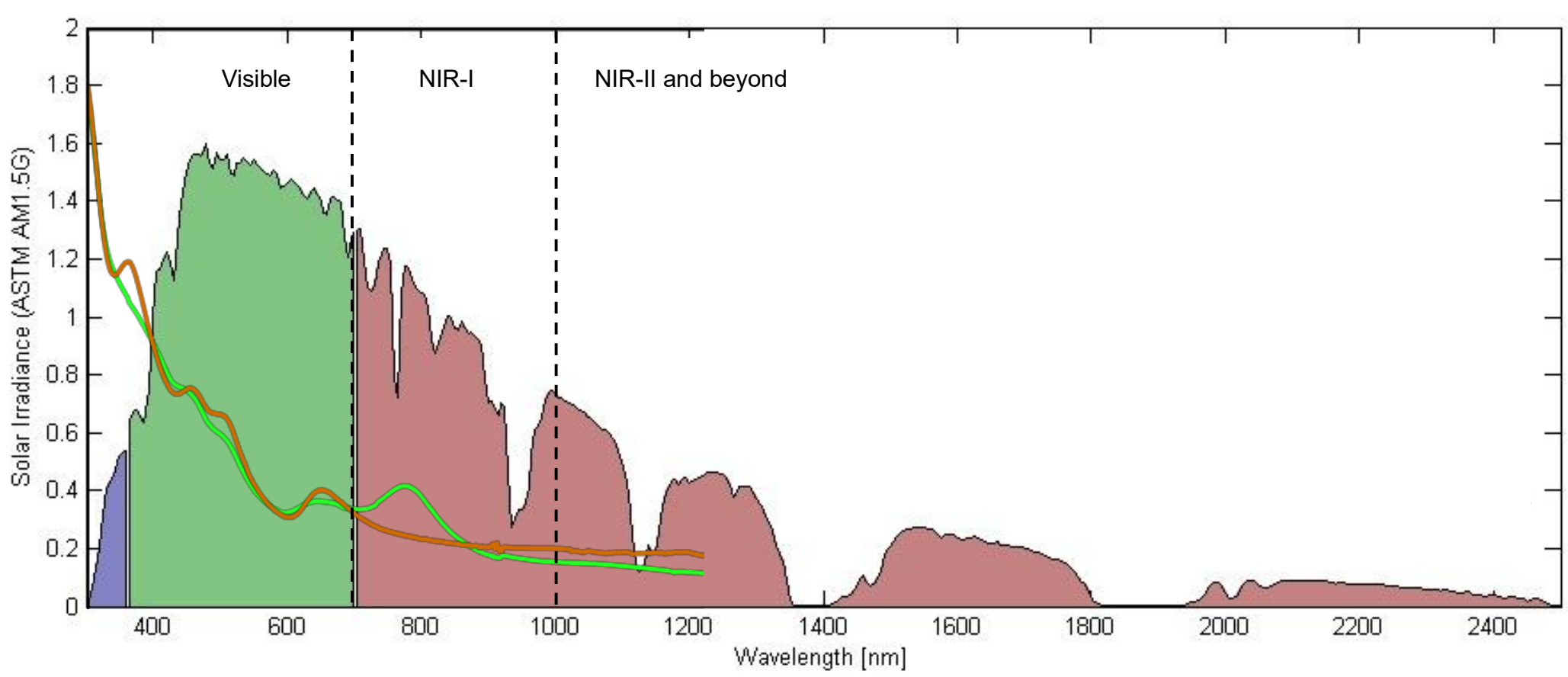

Figure S27. Overlay of AM1.5 Solar spectrum with the UV-vis-NIR spectra of M_{44} (brown) and M_{81} (green) NCs.

S3. SUPPORTING TABLES

Table S1. Photothermal Conversion Efficiency (η) and Corresponding Excitation Wavelengths of Selected Reported Nanomaterials, Focusing on Atomically Precise Nanostructures and Nanoparticles.

Material	Type	Excitation (nm)	η (%)	Ref.
AuNCs@TBN–Superphane	Nanocluster	808	92.8	[8]
**$Au_{43}Ag_{38}$ or M_{81}**	**Nanocluster**	**808**	**91%**	**This work**
Porous Pt Nanoparticles	Nanoparticle	980	90.0	[9]
Au PBs	Plasmonic	808	88.6	[10]
tfm-BDP NPs	Organic NP	808	88.3	[11]
$Au_9(AgCu)_{126}(PhC{\equiv}C)_{68}$	Nanocluster	660	84.7	[12]
Cu_{36}	Nanocluster	445	77.0	[13]
Pt@PDA-c	Nanoparticle	1064	71.3	[14]
$Au_{64}(CPT)_{31}S\cdot$	Nanocluster	660	69.2	[15]
**$Au_{24}Ag_{20}$ or M_{44}**	**Nanocluster**	**808**	**69%**	**This work**
Pt_{23}	Nanocluster	532	68.4	[16]
$(AgCu)_{31}$	Nanocluster	532	67.6	[17]
$Au_{64}(CPT)_{32}$	Nanocluster	980	67.1	[5]
$Au_{44}MBA_{26}$–Cy7	Nanocluster	808	65.1	[18]
$AuAg_{42}$	Nanocluster	606	61.0	[12]
Cu_{62} cluster	Nanocluster	808	56.0	[19]
$Au_{16}Cu_3$-2	Nanocluster	808	55.1	[20]
Pt Spirals	Nanoparticle	1120	52.5	[21]
SPNS	Nanosheet	808	52.0	[22]
Pt/Te nano-heterostructures	Nanoparticle	808	51.8	[23]
$Au_{16}Cu_3$-1	Nanocluster	808	46.5	[20]
$Au_{25}(PET)_{18}$	Nanocluster	660	45.3	[15]
PPy NPs	Organic NP	808	44.7	[24]
$Au_{25}(pMBA)_{18}$	Nanocluster	808	42.2	[25]
$Au_{37}(CHT)_{23}$	Nanocluster	650	42.9	[26]
$Au_{25}(Capt)_{18}$	Nanocluster	808	41.3	[25]
$Au_{25}(AcCys)_{18}$	Nanocluster	808	39.7	[25]
CuNCs (MEA)	Nanocluster	405	38.0	[27]
DA CNDs	Nanodot	808	35.0	[28]
$Au_{66}(PET)_{38}$	Nanocluster	980	34.3	[29]
Ag_{30}	Nanocluster	660	34.6	[30]
$Cu_{29}(CHT)_{18}$	Nanocluster	488	33.0	[31]
$Au_{48}(CHT)_{26}$	Nanocluster	650	30.3	[26]
$Au_{52}(PET)_{32}$	Nanocluster	785	30.2	[29]
Cu_9S_5 nanocrystal	Nanoparticle	980	25.7	[32]
Ag_{34}	Nanocluster	660	22.8	[30]
$Cu_{2-x}Se$ nanocrystal	Nanoparticle	800	22	[33]
$Au_{25}(pMBA)_{18}$–BSA	Nanocluster	808	18.7	[25]
$Au_{25}(SG)_{18}$	Nanocluster	808	16.2	[25]
Au NPs	Nanoparticle	514	10.0	[32]
$Au_{42}(PET)_{32}$	Nanocluster	808	N.R.	[35]
$Ag_{155}(CyS)_{40}(TC4A)_5Cl_2$	Nanocluster	660	N.R.	[36]
Ag_9-NCs/PEI	Nanocluster	660	N.R.	[37]
Cu_{54}	Nanocluster	808	N.R.	[38]
Cu_xAu_{61-x}	Nanocluster	808	N.R.	[39]

Table S2. Atomically precise metal nanoclusters with PL maxima beyond 1300 nm.

NC	Emission Peak (nm)	QY (%)	Ref
**$Au_{43}Ag_{38}(C{\equiv}CR)_{36}Cl_{12}$**	**1320 nm**	**1.5**	**This work**
$Au_{38}(SR)_{24}$	1322 nm	< 2	[40]
$Au_{78}(PET)_{56}$	1400 nm	15.8	[41]
$[Au_{37}(PPh_3)_{10}(PET)_{10}Cl_2]^+$	1520 nm	0.1	[42]
$Au_{96}(PET)_{68}$	1620 nm	1.9	[41]
$Au_{114}(PET)_{80}$	1810 nm	1.0	[41]

SUPPORTING REFERENCES

[1] D. K. Roper, W. Ahn, M. Hoepfner, *J. Phys. Chem. C* **2007**, *111*, 3636–3641.
[2] B. Kim, H. Shin, T. Park, H. Lim, E. Kim, *Advanced Materials* **2013**, *25*, 5483–5489.
[3] W. Gu, Y. Zhou, W. Wang, Q. You, W. Fan, Y. Zhao, G. Bian, R. Wang, L. Fang, N. Yan, N. Xia, L. Liao, Z. Wu, *Angew Chem Int Ed* **2024**, *63*, e202407518.
[4] K. Deng, Y. Chen, C. Li, X. Deng, Z. Hou, Z. Cheng, Y. Han, B. Xing, J. Lin, *J. Mater. Chem. B* **2017**, *5*, 1803–1814.
[5] A. Sardar, Y. Wang, G. He, C. G. Gianopoulos, D. S. N. D. Samarasinghe, Z. Liu, K. Kirschbaum, C. M. Aikens, R. Jin, *ACS Nano* **2026**, *20*, 8827–8837.
[6] J. Xu, L. Xiong, X. Cai, S. Tang, A. Tang, X. Liu, Y. Pei, Y. Zhu, *Chem. Sci.* **2022**, *13*, 2778–2782.
[7] K. Akazawa, S. Ito, Y. Hamasaki, K. Koyasu, T. Tsukuda, *J. Phys. Chem. A* **2025**, *129*, 6043–6048.

(the following refs are for Tables S1 & S2)
[8] Y. Zhang, J. Zhou, K. Luo, W. Zhou, F. Wang, J. Li, Q. He, *Angew. Chem. Int. Ed.* **2025**, *64*, DOI 10.1002/anie.202500058.
[9] X. M. Zhu, H. Y. Wan, H. Jia, L. Liu, J. Wang, *Adv. Healthc. Mater.* **2016**, *5*, 3165.
[10] J. Zhou, Y. Jiang, S. Hou, P. K. Upputuri, D. Wu, J. Li, P. Wang, X. Zhen, M. Pramanik, K. Pu, H. Duan, *ACS Nano* **2018**, *12*, 2643.
[11] D. Xi, M. Xiao, J. Cao, L. Zhao, N. Xu, S. Long, J. Fan, K. Shao, W. Sun, X. Yan, X. Peng, *Advanced Materials* **2020**, *32*, DOI 10.1002/adma.201907855.
[12] N. L. Li, J. Wei, X. Y. Ran, J. Li, L. Shen, F. Zhang, Q. Dai, W. Wang, K. Li, X. K. Wan, *Angew. Chem. Int. Ed.* **2025**, *64*, DOI 10.1002/anie.202503036.
[13] B. Yan, D. S. N. D. Samarasinghe, J. Sun, H. Deng, L. Li, M.-Q. Qi, F. Zhao, Q. Xu, H. Guo, X. Sun, X. Gong, R. Huo, M. Zhu, Q. Wu, Z. Xie, C. Xin, Y. Wang, X. Jiang, S. Li, F. Li, M. Zhou, C. M. Aikens, N. Zheng, H. Shen, *Nat. Commun.* **2026**, DOI 10.1038/s41467-026-70141-8.
[14] S. Qi, Y. Zhang, G. Liu, J. Chen, X. Li, Q. Zhu, Y. Yang, F. Wang, J. Shi, C. S. Lee, G. Zhu, P. Lai, L. Wang, C. Fang, *Acta Biomater.* **2021**, *129*, 245.
[15] Z. Hu, S. Ji, W. Wang, W. Gu, G. Guan, M. Chen, Q. You, Y. Zhou, J. Cheng, L. Liao, N. Yan, Z. Wu, *Angew. Chem. Int. Ed.* **2025**, DOI 10.1002/anie.202517738.
[16] R. Wang, D. Chen, L. Fang, W. Fan, Q. You, G. Bian, Y. Zhou, W. Gu, C. Wang, L. Bai, J. Li, H. Deng, L. Liao, J. Yang, Z. Wu, *Angew. Chem. Int. Ed.* **2024**, *63*, DOI 10.1002/anie.202402565.
[17] L. Fang, W. Fan, G. Bian, R. Wang, Q. You, W. Gu, N. Xia, L. Liao, J. Li, H. Deng, N. Yan, Z. Wu, *Angew. Chem. Int. Ed.* **2023**, *62*, DOI 10.1002/anie.202305604.
[18] G. Yang, X. Mu, X. Pan, Y. Tang, Q. Yao, Y. Wang, F. Jiang, F. Du, J. Xie, X. Zhou, X. Yuan, *Chem. Sci.* **2023**, *14*, 4308.
[19] B. Yan, J. Sun, J. Liu, L. Li, H. Deng, Q. Xu, S. Wang, H. Shen, *JACS Au* **2025**, *5*, 1884.
[20] F. Chen, X. Lei, P. Lan, J. Sun, W. W. Xu, M. Zhou, C. Yao, *Angew. Chem. Int. Ed.* **2026**, DOI 10.1002/anie.4754238.

[21] Q. Wang, H. Wang, Y. Yang, L. Jin, Y. Liu, Y. Wang, X. Yan, J. Xu, R. Gao, P. Lei, J. Zhu, Y. Wang, S. Song, H. Zhang, *Advanced Materials* **2019**, *31*, DOI 10.1002/adma.201904836.
[22] S. Tang, M. Chen, N. Zheng, *Small* **2014**, *10*, 3139.
[23] C. Q. Li, X. L. Hou, D. X. Jiang, B. Zhang, M. W. Ma, X. T. Xie, Y. Di Zhao, T. C. Liu, B. Liu, *ACS Sustain. Chem. Eng.* **2022**, *10*, 16598.
[24] M. Chen, X. Fang, S. Tang, N. Zheng, *Chemical Communications* **2012**, *48*, 8934.
[25] S. Kundu, H. Yuan, R. Antoine, *Journal of Physical Chemistry Letters* **2025**, *16*, 8480.
[26] F. Jin, H. Dong, Y. Zhao, S. Zhuang, L. Liao, N. Yan, W. Gu, J. Zha, J. Yuan, J. Li, H. Deng, Z. Gan, J. Yang, Z. Wu, *Acta Chimi. Sin.* **2020**, *78*, 407.
[27] A. Ferlazzo, S. Bonforte, F. Florio, S. Petralia, L. Sorace, B. Muzzi, A. Caneschi, A. Gulino, *Mater. Adv.* **2024**, *5*, 8034.
[28] Y. Li, X. Zhang, M. Zheng, S. Liu, Z. Xie, *RSC Adv.* **2016**, *6*, 54087.
[29] W. Gu, Y. Zhou, W. Wang, Q. You, W. Fan, Y. Zhao, G. Bian, R. Wang, L. Fang, N. Yan, N. Xia, L. Liao, Z. Wu, *Angew. Chem. Int. Ed.* **2024**, *63*, DOI 10.1002/anie.202407518.
[30] Z. Wang, H. Zhao, Y. Z. Li, C. Zhang, R. K. Gupta, C. H. Tung, D. Sun, *Nano Lett.* **2024**, *24*, 458.
[31] A. Sardar, Y. Wang, A. Mazumder, G. He, C. G. Gianopoulos, K. Kirschbaum, R. Jin, *Inorg. Chem.* **2025**, *64*, 17687.
[32] Q. Tian, F. Jiang, R. Zou, Q. Liu, Z. Chen, M. Zhu, S. Yang, J. Wang, J. Wang, J. Hu, *ACS Nano* **2011**, *5*, 9761.
[33] C. M. Hessel, V. P. Pattani, M. Rasch, M. G. Panthani, B. Koo, J. W. Tunnell, B. A. Korgel, *Nano Lett.* **2011**, *11*, 2560.
[34] D. K. Roper, W. Ahn, M. Hoepfner, *Journal of Physical Chemistry C* **2007**, *111*, 3636.
[35] Y. Li, Y. Song, X. Zhang, T. Liu, T. Xu, H. Wang, D. E. Jiang, R. Jin, *J. Am. Chem. Soc.* **2022**, *144*, 12381.
[36] Z. Wang, F. Alkan, C. M. Aikens, M. Kurmoo, Z. Y. Zhang, K. P. Song, C. H. Tung, D. Sun, *Angew. Chem. Int. Ed.* **2022**, *61*, DOI 10.1002/anie.202206742.
[37] Y. Wang, C. Zhang, T. Xu, Y. Li, H. Xie, D. Sun, N. Feng, S. Li, X. Xin, *ACS Appl. Mater. Interfaces* **2025**, *17*, 17307.
[38] X. Sun, B. Yan, X. Gong, Q. Xu, Q. Guo, H. Shen, *Chemistry - A European Journal* **2024**, *30*, DOI 10.1002/chem.202400527.
[39] T. Xu, J. Kong, Y. Chen, W. Cui, Y. Fang, W. Zhang, M. Wang, M. Zhou, Y. Li, R. Jin, Y. Song, *Advanced Science* **2025**, *12*, DOI 10.1002/advs.202509283.
[40] Z. Liu, Y. Wang, W. Ji, X. Ma, C. G. Gianopoulos, S. Calderon, T. Ma, L. Luo, A. Mazumder, K. Kirschbaum, E. C. Dickey, L. A. Peteanu, D. Alfonso, R. Jin, *ACS Nano* **2025**, *19*, 9121–9131.
[41] L. Luo, G. He, Z. Liu, A. Sardar, W. Ji, A. Mazumder, S. Chen, Q. Li, R. Jin, *J. Am. Chem. Soc.* **2026**, *148*, 12520–12525.
[42] Q. Li, C. J. Zeman, Z. Ma, G. C. Schatz, X. W. Gu, *Small* **2021**, *17*, 2007992.
